# Structural insights into plasmalemma vesicle-associated protein (PLVAP): Implications for vascular endothelial diaphragms and fenestrae

**DOI:** 10.1073/pnas.2221103120

**Published:** 2023-03-30

**Authors:** Tao-Hsin Chang, Fu-Lien Hsieh, Xiaowu Gu, Philip M. Smallwood, Jennifer M. Kavran, Sandra B. Gabelli, Jeremy Nathans

**Affiliations:** ^a^Department of Molecular Biology and Genetics, Johns Hopkins University School of Medicine, Baltimore, MD 21205; ^b^HHMI, Johns Hopkins University School of Medicine, Baltimore, MD 21205; ^c^Department of Biophysics and Biophysical Chemistry, Johns Hopkins University School of Medicine, Baltimore, MD 21205; ^d^Department of Biochemistry and Molecular Biology, Bloomberg School of Public Health, Johns Hopkins University, Baltimore, MD 21205; ^e^Department of Oncology, Johns Hopkins University School of Medicine, Baltimore, MD 21205; ^f^Department of Medicine, Johns Hopkins University School of Medicine, Baltimore, MD 21205; ^g^Department of Neuroscience, Johns Hopkins University School of Medicine, Baltimore, MD 21205; ^h^Wilmer Eye Institute, Johns Hopkins University School of Medicine, Baltimore, MD 21205

**Keywords:** vasculature, coiled-coil, single-wavelength anomalous dispersion of sulfur atoms, permeability, alpha helix

## Abstract

In many organs, small openings across capillary endothelial cells (ECs) allow the diffusion of low–molecular weight compounds and small proteins between the blood and tissue spaces. These openings contain a diaphragm composed of radially arranged fibers, and current evidence suggests that a single-span type II transmembrane protein, plasmalemma vesicle-associated protein-1 (PLVAP), constitutes these fibers. Here, we present the three-dimensional crystal structure of an 89-amino acid segment of the PLVAP extracellular domain (ECD) and show that it adopts a parallel dimeric alpha-helical coiled-coil configuration with five interchain disulfide bonds. The structure was solved using single-wavelength anomalous diffraction from sulfur-containing residues (sulfur SAD) to generate phase information. Biochemical and circular dichroism (CD) experiments show that a second PLVAP ECD segment also has a parallel dimeric alpha-helical configuration—presumably a coiled coil—held together with interchain disulfide bonds. Overall, ~2/3 of the ~390 amino acids within the PLVAP ECD adopt a helical configuration, as determined by CD. We also determined the sequence and epitope of MECA-32, an anti-PLVAP antibody. Taken together, these data lend strong support to the model of capillary diaphragms formulated by Tse and Stan in which approximately ten PLVAP dimers are arranged within each 60- to 80-nm-diameter opening like the spokes of a bicycle wheel. Passage of molecules through the wedge-shaped pores is presumably determined both by the length of PLVAP—i.e., the long dimension of the pore—and by the chemical properties of amino acid side chains and N-linked glycans on the solvent-accessible faces of PLVAP.

## Significance

The microscopic openings across the walls of many capillaries contain a protein mesh that is formed by the extracellular domain (ECD) of plasmalemma vesicle-associated protein (PLVAP). The three-dimensional structure of one segment of the PLVAP ECD together with biochemical and spectroscopic experiments on this and other segments show that the majority of the PLVAP ECD consists of a dimeric alpha-helical coiled coil, consistent with electron micrographs showing thin fibrous strands arranged across the capillary openings like the spokes of a bicycle wheel. Protein structure determination benefited from the availability of a microfocused long-wavelength X-ray beam, a helical data collection strategy, and an ultrasensitive detector, which together allowed rapid rotation and translation of the crystal during collection of anomalous scattering data from sulfur atoms.

The efficient movement of molecules between the intravascular space and the surrounding tissues is central to vascular function (1). Movement of molecules across capillary endothelial cells (ECs) can occur via a paracellular (between cells) pathway or via any of several transcellular pathways (1). The transcellular pathways include i) transporter- or channel-mediated uptake or release of small molecules (amino acids, glucose, ions, xenobiotics, etc.), ii) receptor-mediated endocytosis, as seen for iron uptake by the transferrin receptor, and iii) diffusion of small- and moderately sized molecules, such as peptide hormones, across 60- to 80-nm circular openings at the endothelial cell plasma membrane (1–3). These circular openings can either span the width of the cell where the EC is extremely thin (fenestrae) or they can serve as the mouth of transendothelial channels or transcytotic vesicular carriers (caveolae), where the EC is thicker (4). The relative prominence of these different pathways differs among vascular beds in different tissues (5, 6).

**Fig. 1.**
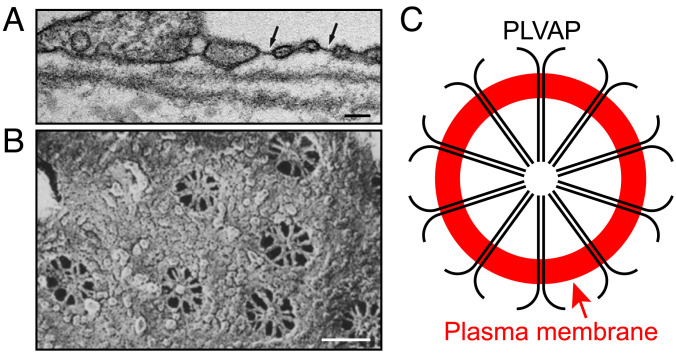
Fenestral diaphragms visualized by electron microscopy and a schematic of PLVAP within a single diaphragm. (*A*) Transmission electron micrograph of a part of a choriocapillaris EC in cross-section showing several fenestrae, each with a single diaphragm (dark line). Two fenestrae are marked with arrows. Reproduced with permission from Hernnberger et al (10). (*B*) Scanning electron micrograph of a part of the luminal face of a rat kidney EC showing an en face view with seven fenestrae. Radial strands that constitute the diaphragm are seen within each fenestra. Reproduced with permission from Bearer and Orci (7). (Scale bars for (*A*) and (*B*), 100 nm.) (*C*) Diagram illustrating a model for the arrangement of PLVAP homodimers within an EC diaphragm. Each black line represents a single PLVAP monomer. Ten dimers are arranged within a circle formed by the plasma membrane (in red).

In the electron microscope, the 60- to 80-nm openings are seen to possess a delicate diaphragm with a thickness of tens of nanometers (Fig. 1*A*). In en face views (Fig. 1 *B* and *C*), the diaphragm is observed to consist of 8 to 10 radial strands joined together at a central hub, like the spokes of a bicycle wheel (7). Current evidence suggests that a single transmembrane protein, plasmalemma vesicle-associated protein (PLVAP or PV1), constitutes the diaphragm’s sole structural protein (8). PLVAP is a type II transmembrane protein of ~60 kDa with an ~390-amino acid C-terminal extracellular domain (ECD), a single transmembrane domain, and a 26-amino acid N-terminal cytoplasmic domain (9). PLVAP localizes to diaphragms by immunoelectron microscopy, and targeted disruption of the mouse *Plvap* gene leads to loss of diaphragms from fenestrae, transendothelial channels, and caveolae (10, 11). Current models envision a diffusional pathway consisting of i) the spaces between PLVAP strands within the diaphragm and ii) a meshwork of N-linked glycans on the PLVAP extracellular domain (4). Additionally, PLVAP functions as a gatekeeper for lymphocyte transmigration through ECs (12) and for the entry of lymphocytes into lymph nodes (13). In gut ECs, *Salmonella typhimurium* has been found to induce the expression of PLVAP, promoting vascular dissemination of gut antigens (14).

PLVAP self-associates based on coimmunoprecipitation experiments with epitope-tagged PLVAP produced in transfected cells, and that association appears to represent a disulfide-linked dimer based on the electrophoretic mobility of PLVAP in the presence vs. absence of reducing agents (15, 16). These data, together with computational analyses of the secondary structure that predict high alpha-helical content for the PLVAP ECD, have led to a model in which this domain forms an extended and largely helical homodimer that spans the ~30- to 40-nm distance from the plasma membrane to the central hub (4). As the ECD contains nine cysteines, the dimer may be held together by multiple interchain disulfide bonds.

To understand how endothelial diaphragms determine vascular permeability, it is essential to define the three-dimensional structure of PLVAP at atomic resolution. Prior to the present study, there were no biophysical studies of purified PLVAP or its fragments, and there was no high-resolution structural information for PLVAP. Here, we present circular dichroism (CD) analyses of the PLVAP ECD and its fragments showing that these polypeptides are almost entirely alpha-helical, electrophoretic analyses of secreted PLVAP fragments with distinct tags showing that they form parallel dimers, and an atomic resolution X-ray structure of an 89-amino acid fragment of the PLVAP ECD showing that it consists of an extended alpha-helical homodimer, held together by five disulfide bonds. The structure was solved using single-wavelength anomalous diffraction from sulfur-containing amino acids (sulfur SAD). We also demonstrate the utility of combining sulfur SAD with molecular replacement and protein modeling for coiled-coil structure determination. Finally, we map the epitope of MECA-32, an anti-PLVAP monoclonal antibody (mAb) (15), and determine the sequences of the MECA-32 light- and heavy-chain variable domains.

## Results

### PLVAP ECD Adopts a Predominantly Alpha-Helical Configuration.

The secondary structure predictions for the PLVAP ECD using the Quick2D program (17) which contains five algorithms (PsiPred, SPIDER3, PSSpred, DeepCNF, and NetSurfP-2.0) show high alpha-helical propensity [~91% helicity (*SI Appendix*, Fig. S1*A*)]. Two regions spanning amino acids 141-224 and 272-389 are predicted to be in a coiled-coil (CC) configuration by MARCOIL (17) and DeepCoil (18), and we, therefore, refer to these as CC1 and CC2, respectively (*SI Appendix*, Fig. S1). To express biophysical quantities of the full-length PLVAP ECD and the individual CC1 and CC2 subregions in a soluble and correctly folded and disulfide-bonded form, PLVAP coding region sequences were inserted downstream of DNA sequences coding for *E. coli* thioredoxin, an 8xHis tag, and the cleavage site for 3C protease (*SI Appendix*, Figs. S2 *A* and *B* and S3 *A* and *B*). Protein production in *E. coli* SHuffle cells, in which the intracellular redox potential favors disulfide bond formation (19), led to the accumulation of the predicted fusion proteins as the major soluble polypeptides (*SI Appendix*, Figs. S2 *C* and S3 *C*). Following immobilized metal affinity chromatography (IMAC) purification, cleavage with 6xHis-tagged 3C protease, and removal of the His-tagged thioredoxin and 3C protease, the PLVAP ECD and its subregions appeared to be present as disulfide-linked dimers based on their electrophoretic mobilities in reducing and nonreducing gels (*SI Appendix*, Figs. S2 *C* and *D* and S3 *C* and *D*).

**Fig. 2.**
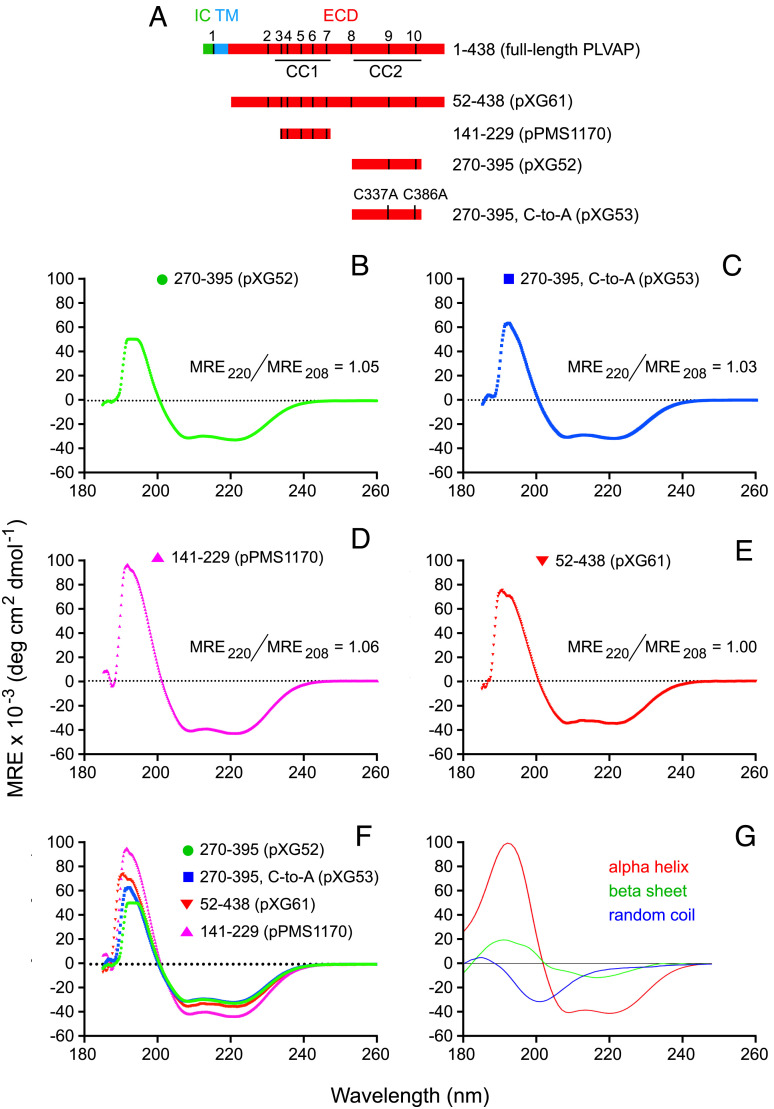
Circular dichroism (CD) spectra of the PLVAP ECD show predominantly alpha-helical content. (*A*) Map of full-length PLVAP (*Top*) and the four soluble ECD fragments analyzed by CD (*Below*). The 10 cysteines are numbered and represented by vertical black lines in the full-length schematic. (*B*–*E*), CD spectra of the indicated ECD segments. (*F*) Superposition of the four CD spectra. (*G*) Reference CD spectra for alpha-helix, beta-sheet, and random coil.

To assess the secondary structure content of the PLVAP ECD, CD spectra were obtained from IMAC-purified soluble dimeric ECD segments encompassing residues 52 to 438 (for the full-length ECD), 141 to 229 (for CC1), and 270 to 395 (for CC2) (Fig. 2). A derivative of CC2, in which both cysteines were mutated to alanine, was also analyzed. The K2D3 algorithm (20) was used to analyze the CD spectra (Fig. 2 *B*–*G*), revealing ~90% helicity for CC1 (pPMS1170), ~74 to 78% helicity for CC2 (irrespective of the presence or absence of the two cysteines, pXG52 and pXG53, respectively), and ~64% helicity for the full-length PLVAP ECD (pXG61), lower than the predicted ~91% helicity (*SI Appendix*, Fig. S1*A*).

### PLVAP ECD Forms Parallel Disulfide-Linked Dimers.

**Fig. 3.**
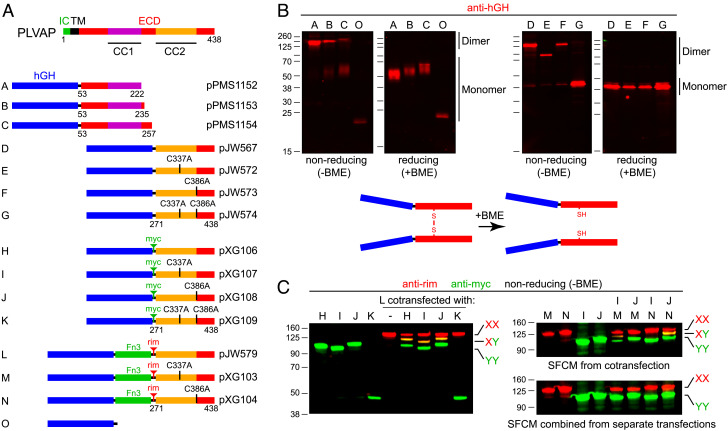
Biochemical evidence that PLVAP ECD fragments form parallel homodimers linked by disulfide bonds between corresponding cysteines. (*A*) Map of full-length PLVAP (*Top*) and secreted human growth hormone (hGH; blue rectangle) fusions with the indicated ECD fragments (*Below*). The CC2 region of PLVAP has two cysteines at positions 337 and 386, and various derivatives have either one or both mutated to alanine, as indicated for the fusion proteins labeled D to N. The tenth fibronectin type 3 (Fn3) domain from human fibronectin (amino acids 1,538 to 1,630) is shown as a green rectangle in fusion proteins L, M, and N. (*B*) SDS-PAGE and anti-hGH immunoblots of serum-free conditioned medium from transiently transfected HEK/293T cells showing the electrophoretic mobilities of the indicated fusion proteins under either reducing [+beta-mercaptoethanol (BME)] or nonreducing [-BME] conditions. Fusion proteins D, E, and F have identical lengths but exhibit distinct electrophoretic mobilities under nonreducing conditions based on the location of the disulfide bonds. Fusion protein G, which lacks interchain disulfide bonds, migrates as a monomer after heating in SDS in the absence of BME. D, dimer. M, monomer. (*C*) SDS-PAGE and anti-epitope tag immunoblots of serum-free conditioned medium (SFCM) showing that coexpression and cosecretion of pairs of PLVAP ECD fusion proteins with different electrophoretic mobilities and different epitope tags lead to both homo- and heterodimer formation. The dimers are stable to heating in SDS in the absence of BME if there is at least one interchain disulfide bond. Heterodimers do not form if SFCM containing the individual homodimeric fusion proteins are mixed together prior to gel electrophoresis. Immunoblotting was performed with mouse anti-rim mAb and rabbit anti-myc, and the primary antibodies were visualized with fluorescent secondary antibodies.

To explore the assembly of the PLVAP ECD in a more native context, segments corresponding to its N- and C-terminal halves were expressed as secreted fusions to human growth hormone (hGH) in transiently transfected HEK/293T cells and harvested from serum-free conditioned medium (SFCM). A set of PLVAP ECD N-terminal regions, encompassing amino acids 53-222, 53-235, and 53-257 (constructs A-C, respectively, in Fig. 3*A*), all of which include CC1, were efficiently secreted and found to migrate in nonreducing SDS-PAGE at a mobility corresponding to 2 to 3 times the molecular weight at which they migrate in reducing SDS-PAGE (Fig. 3*B*). hGH alone (construct O) migrates with a predicted molecular weight of ~20 kDa under both reducing and nonreducing conditions, consistent with its monomeric structure.

The C-terminal half of the PLVAP ECD, amino acids 271-438, encompasses CC2 and contains two cysteines. In addition to the wild-type (WT) version of this region, site-directed mutants with the first cysteine changed to alanine (C337A) and the second cysteine changed to alanine (C386A), or both cysteines changed to alanine were produced as hGH fusions (constructs D-G in Fig. 3*A*). In reducing SDS-PAGE, all four hGH fusion proteins migrated at the same mobility, consistent with their monomeric molecular weights (~40 kDa; Fig. 3*B*). However, under nonreducing conditions, only the cysteine-to-alanine double mutant migrated at the monomeric molecular weight, whereas the three mutants that contain either one or two cysteines exhibited lower mobility. Under nonreducing conditions (Fig. 3*B*), the C337A mutant (construct E) shows a higher mobility than the other two cysteine-containing fusion proteins (constructs D and F), presumably reflecting a distinct conformation of its unfolded state. In sum, the electrophoretic mobilities of hGH–PLVAP fusion proteins A-G under reducing vs. nonreducing conditions imply that both the N-terminal and C-terminal halves of the PLVAP ECD form disulfide-linked dimers.

To more comprehensively probe the dimeric nature of the C-terminal half of the PLVAP ECD, we prepared constructs H–K, a set of myc epitope–tagged versions of constructs D–G, and constructs L–N, a set of rim epitope-tagged and fibronectin type 3 (Fn3) domain-containing versions of constructs D–F. Based on their distinct epitope tags, members of these two sets of constructs can be independently visualized in fluorescent immunoblots. Additionally, the higher molecular weight of constructs L–N lowers their mobility in nonreducing gels. Heterodimers formed between individual members of constructs H–K and constructs L–N would be predicted to exhibit a mobility distinct from and intermediate to the mobilities of the corresponding homodimers. In the immunoblots of nonreducing SDS-PAGE gels shown on the left side of Fig. 3*C*, SFCM harvested from cells cotransfected with construct L together with constructs H, I, J, or K reveals L+L homodimers (red; low mobility), H+H, I+I, and J+J homodimers (green; high mobility), and L+H, L+I, and L+J heterodimers (yellow; intermediate electrophoretic mobility). Consistent with the behavior of construct G (Fig. 3*B*), cotransfection of constructs L and K shows that L+K heterodimers and K+K homodimers are unstable to heating in SDS.

To further explore the disulfide bonding arrangement within PLVAP CC2, we tested the stabilities of heterodimers formed between all pairwise combinations of constructs I and J cotransfected with constructs M and N. In each of these constructs, one of the two cysteines has been mutated to alanine. If the presumptive dimers are aligned in a parallel fashion and if disulfide bonding is intermolecular, we would predict that only the pairs of constructs in which the same cysteine is preserved would form disulfide-linked heterodimers, i.e., I+M and J+N. By contrast, if the presumptive dimers are aligned in an anti-parallel fashion and if disulfide bonding is intermolecular, we would predict that only the pairs of constructs in which different cysteines are preserved would form disulfide-linked heterodimers, i.e., I+N and J+M. As seen in the upper immunoblot on the right side of Fig. 3*C*, only the SFCM harvested from cells cotransfected with constructs I+M and J+N shows the heterodimeric yellow band at intermediate mobility. As a further control for specificity, the lower immunoblot on the right side of Fig. 3*C* shows that pairwise mixing of SFCM from cells transfected with individual constructs in the same combinations as shown for the cotransfection experiment does not generate heterodimer species. This experiment implies that dimers of the C-terminal half of the PLVAP ECD are likely formed intracellularly and do not rapidly exchange their subunits at room temperature. Taken together, these experiments show that CC2 forms a parallel dimer with two intermolecular disulfide bonds.

### Protein Production and Crystallization of PLVAP CC1 and CC2.

For structure determination, we focused on PLVAP CC1 and CC2. Both protein fragments can be expressed as soluble disulfide-linked dimers as judged by their mobilities on reducing vs. nonreducing SDS-PAGE gels, and both are monodisperse as judged by their behavior in size-exclusion chromatography (SEC; *SI Appendix*, Figs. S2 and S3). PLVAP CC2 crystallized in several conditions (e.g., 0.1 M MgCl_2_, 0.1 M MES, pH 6.0, and 8% polyethylene glycol 6K), and the crystals diffracted to a resolution of 3.65 Å, revealing an *I*2_1_2_1_2_1_ space group with unit-cell parameters 82.6 Å, 98.2 Å, and 413.6 Å. Of note is that PLVAP CC2 crystals revealed a severely anisotropic diffraction pattern, with resolution limits along to a*, b*, and c* axes of 6.6 Å, 5.3 Å, and 3.65 Å. Moreover, each asymmetric unit appears to contain 4 to 5 dimers of PLVAP CC2 based on an analysis of the Matthews coefficient (21). Without an accurate model for molecular replacement, and with low-resolution and severe anisotropic diffraction data (22), we were unable to solve the structure of this crystal form.

PLVAP CC1 crystallized in the *I*2_1_2_1_2_1_ space group with unit-cell parameters 33.8 Å, 89.0 Å, and 180.9 Å, referred to hereafter as crystal form II. X-ray diffraction data were collected and processed to 1.95 Å (*SI Appendix*, Fig. S4 *A*–*C* and Table S1). Because PLVAP CC1 contains only one methionine, we initially produced PLVAP CC1 with selenium-labeled methionine (SeMet) for multi- or single-wavelength anomalous diffraction (MAD or SAD). However, the resulting anomalous signals were too weak to be useful for structure determination.

### Structure Determination of PLVAP CC1 by Sulfur SAD Phasing.

**Fig. 4.**
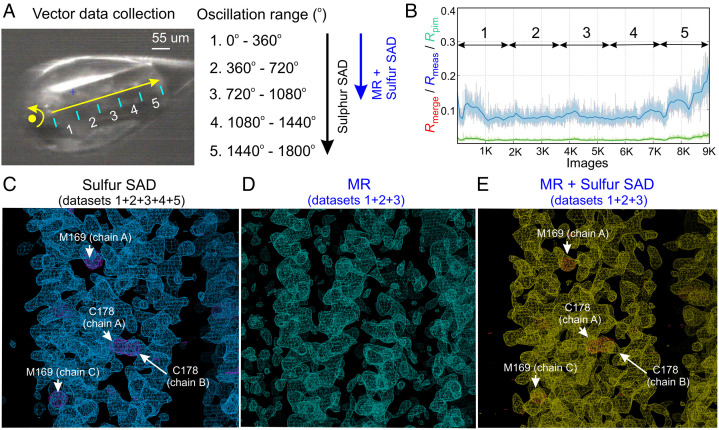
Data collection and phasing methods to determine the structure of PLVAP CC1. (*A*) The vector module established on the AMX beamline (NSLS II) was used to collect a continuous 1,800° oscillation range (9,000 images) along 300 μm of a single PLVAP CC1 crystal (*P*2_1_2_1_2_1_ space group). For the phase determination and analysis, every 360° rotation was scaled as a single dataset (1 to 5) using AIMLESS. (*B*) The plot shows the data quality for each image. The *R*_merge_ (red lines) are not visible on the plot because they are completely overlapping with *R*_meas_ (blue lines). Each 360° rotation is denoted by double-headed arrows. (*C*) The initial density-modified map (blue meshes) from PHENIX RESOLVE was calculated with experimental sulfur SAD phases from five datasets and is contoured at 1.3 σ. The anomalous difference map (purple meshes) for sulfur atoms is contoured at 3 σ with sulfur-containing residues labeled. (*D*) The initial electron density map (cyan meshes) from PHASER contoured at 1.3 σ, was obtained by MR using the computational model of PLVAP CC1 generated by the CCFold algorithm as the search model. (*E*) The initial density-modified map (yellow meshes) from PHENIX RESOLVE was calculated with the phases obtained by combining MR with sulfur SAD using only the first three datasets and is contoured at the 1.3 σ level. The anomalous difference map (red meshes) for sulfur atoms is contoured at the 3 σ level with sulfur-containing residues labeled.

PLVAP CC1 contains five cysteines and one methionine, and previous studies have demonstrated that SAD from sulfur-containing residues—sulfur SAD—can be used for experimental phasing and structure determination (23–26). The recent development and installation of a microfocused X-ray beam, fast pixel array detector, and stable vector/helical data collection system at the National Synchrotron Light Source II (NSLS II) inspired us to ask whether it might be possible to collect accurate sulfur SAD data from a single PLVAP CC1 crystal for de novo phasing. For this experiment, we first screened additional crystallization conditions and obtained PLVAP CC1 crystals in the *P*2_1_2_1_2_1_ space group with unit-cell parameters 35.6 Å, 86.2 Å, and 181.6 Å, referred to hereafter as crystal form I. We then collected data at a wavelength of 1.77 Å, using a low-noise and fast readout EIGER X detector, with an exposure time of 0.015 s per 0.2° oscillation. The X-ray beam was 90% attenuated to give ~5 × 10^11^ photons s^−1^ with a beam cross-section of 5 μm × 7 μm. The data collection encompassed a total rotation of 1,800° while continuously advancing the crystal 300 μm (Fig. 4*A*). Constraints related to the detector-to-crystal distance and the long wavelength of the X-ray beam limited the resolution of the sulfur SAD data to 2.4 Å (*SI Appendix*, Fig. S4 *D*–*F*).

**Fig. 5.**
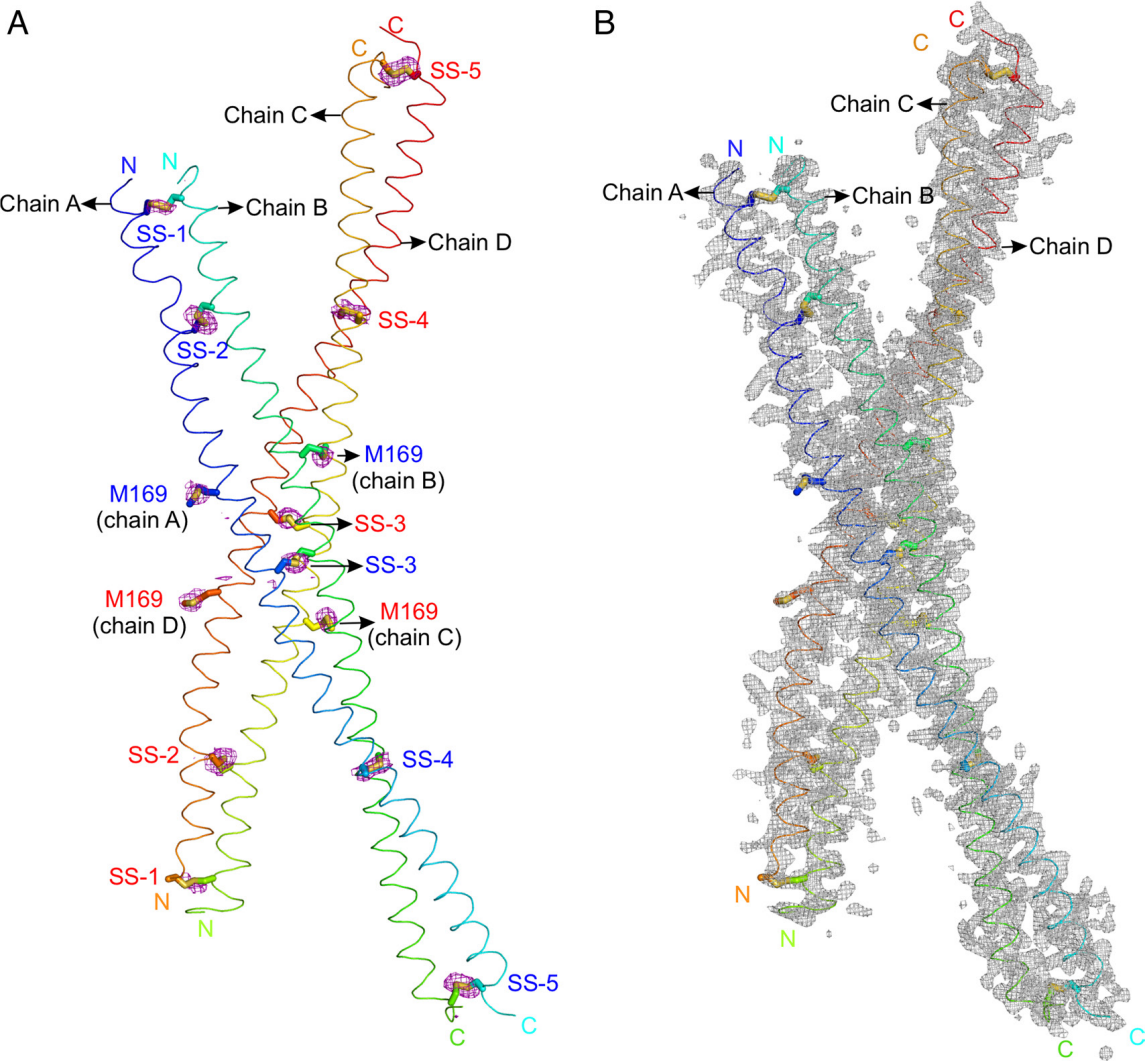
Structure determination of PLVAP CC1 (crystal form I) using experimental sulfur SAD phases. (*A*) The anomalous difference map (purple meshes) for sulfur atoms, contoured at 2.5 σ, is shown with two dimers of PLVAP CC1 in the asymmetric unit of crystal form I (*P*2_1_2_1_2_1_ space group) in a ribbon representation. Five disulfide bonds (SS-1 to SS-5) and two methionine sulfur atoms (M169) can be identified in each CC1 dimer using sulfur SAD phases. The N and C termini are labeled. (B) The sigmaA-weighted 2|*F*_O_|-|*F*_C_| electron density map is shown after structure refinement (gray meshes) and is contoured at 1.0 σ.

To determine how much data are required for sulfur SAD phasing of PLVAP CC1, we indexed and integrated the complete 1,800° dataset and then divided it into five separately scaled datasets, each comprising one 360° rotation (Fig. 4 *A* and *B*). We found that integrating all five sulfur SAD datasets provided anomalous signals with a signal-to-noise ratio sufficient for structure determination of PLVAP CC1, whereas integrating only the first three sulfur SAD datasets was insufficient (Fig. 4*C*; compare *SI Appendix*, Figs. S5 and S6). Phases derived from the full sulfur SAD dataset revealed electron densities corresponding to ten disulfide bonds and four methionines (Fig. 5*A*). The Bijvoet diffraction ratio ( $<|{∆F}_{\pm h}|>\;/\;<|F|>$ ) was ~1.4% at 1.77 Å (23). Subsequent structure refinement revealed two PLVAP CC1 dimers in the asymmetric unit (Fig. 5*B* and *SI Appendix*, Table S1).

### Integrating Sulfur SAD with Structure Prediction and Molecular Replacement.

The past several years have witnessed dramatic advances in protein structure prediction (27, 28), which makes structure determination using molecular replacement (MR) based on predicted structures an increasingly attractive strategy. Although predicted structures can capture the overall secondary and tertiary structural features of the protein of interest, they often exhibit multiple differences from the actual structure on a scale of one to several angstroms. As a result, phases generated by MR based on predicted structures can be of variable utility. One approach to improving MR phasing is to combine it with SAD phasing (MR–SAD) (29–32). This strategy has not yet been applied to coiled-coil structures. Therefore, we have explored the utility of this approach with PLVAP CC1 as a test case.

For this test, we first combined the X-ray diffraction data from the first three crystal rotations corresponding to 3 × 360 ^o^ (datasets 1 + 2 + 3) = 1,080°, as described in the preceding section (Fig. 4 *A* and *B*). As noted above, phases calculated from this 1,080° sulfur SAD dataset failed to solve the structure of PLVAP CC1 (*SI Appendix*, Fig. S6). Next, we used the CCFold algorithm (33), a protein structure prediction algorithm for coiled-coil proteins, to generate a computational model of PLVAP CC1. This model has a rmsd of 1.5 Å relative to the crystal structure of PLVAP CC1, with the N-terminal region showing the largest deviation (*SI Appendix*, Fig. S7). We used this computational model for MR with the 1,080° diffraction dataset to generate an electron density map that exhibited a coiled-coil-like pattern (Fig. 4*D* and *SI Appendix*, Fig. S8*A*). However, this approach appeared to retain an inherent MR model bias, with the result that the MR-based phases were of insufficient quality to permit structure refinement beyond values of *R*_work_ = 46.8% and *R*_free_ = 50.4% (Fig. S8*B*).

To test the MR–SAD strategy, we used the MR-based electron density map and its protein model in combination with the sulfur SAD phases from the 1,080° diffraction dataset to improve the anomalous difference Fourier analyses. Subsequent automated model building and structure refinement from this starting point resulted in successful structure determination with *R*_work_ = 31.7% and *R*_free_ = 37.3% (Fig. 4*E* and *SI Appendix*, Fig. S8*C*). During the course of this work, the MR–SAD pipeline in PHENIX AutoSol (34, 35) was updated to improve anomalous difference Fourier analyses by integrating the model and the electron density map obtained from MR. Taken together, these tests show that the combination of protein modeling and MR–SAD can successfully solve a coiled-coil structure in a situation where phases based on modeling and MR alone or sulfur SAD alone cannot.

### Structure of PLVAP CC1.

**Fig. 6.**
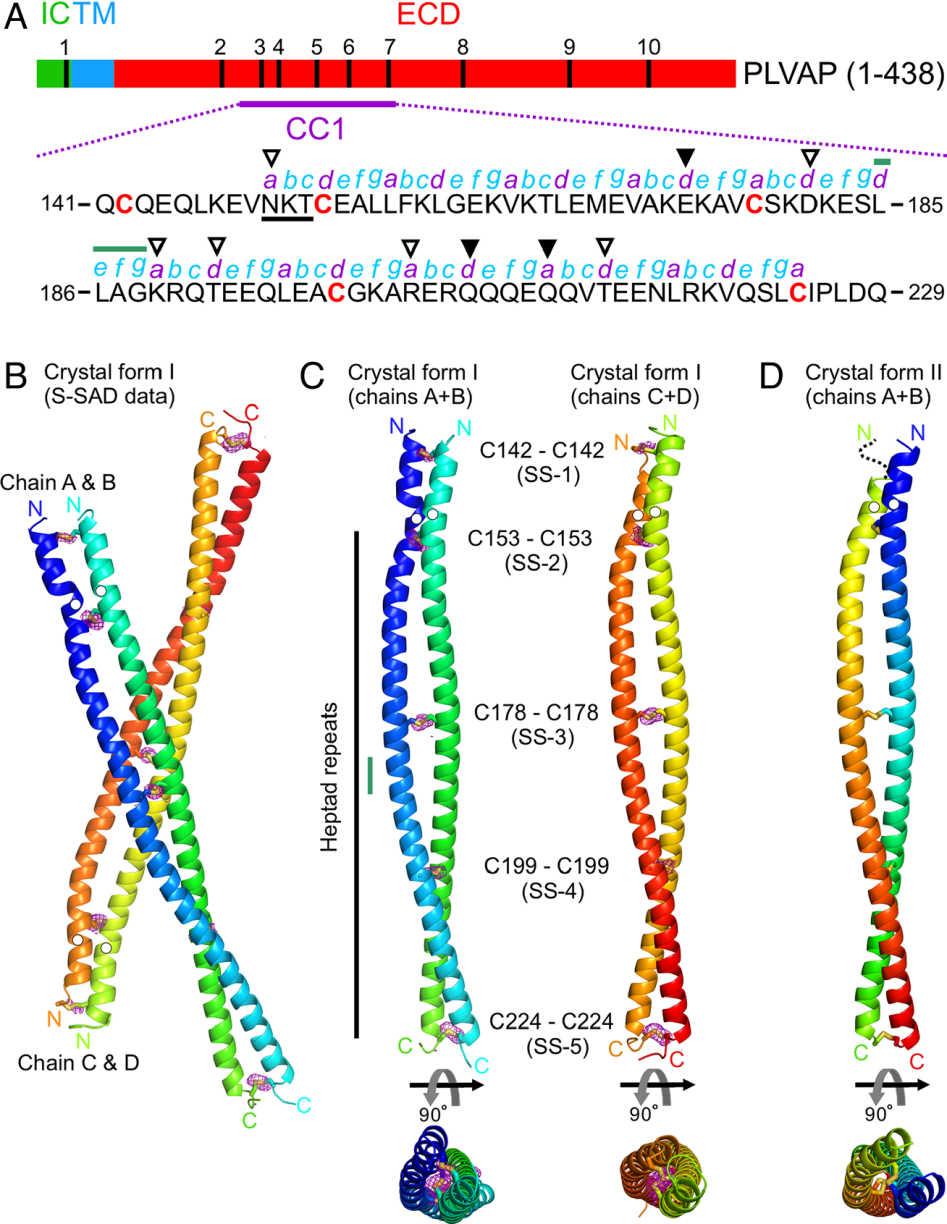
Structures of PLVAP CC1. (*A*) Schematic diagram of mouse PLVAP primary structure showing the intracellular domain (IC), the transmembrane domain (TM), the extracellular domain (ECD), and the location and sequence of PLVAP CC1 (residues 141 to 229). The five cysteine residues in CC1 are denoted in red bold letters. The putative N-linked glycosylation site (Asn-X-Ser/Thr; X is any residue) is indicated by black underline. The heptad repeat pattern (*abcdefg*; *a* and *d* are generally hydrophobic residues) is labeled and was derived by the Socket2 algorithm (36) based on the structures of PLVAP CC1. The unfilled triangles indicate amino acids at positions *a* and *d* that are hydrophilic (Asn150, Asp181, Lys189, Thr192, Arg203, and Thr213) and contribute to coiled-coil interactions (Fig. 7). The filled triangles indicate amino acids at positions *a* and *d* (Glu174, Gln206, and Gln210) that do not appear to contribute to coiled-coil interactions. The green line indicates an insertion of a noncanonical heptad repeat (*defg*). In this region, disulfide bonds SS-3 and SS-4 likely maintain the coiled-coil configuration. (*B* and *C*) Crystal form I of PLVAP CC1 solved by sulfur SAD. The anomalous difference map (purple meshes) contoured at 3.0 σ confirms the presence of five disulfide bonds (SS-1 to SS-5) shown in stick representation. The N and C termini are labeled. The positions of Asn residues in the putative N-linked glycosylation sites are indicated by white circles. In (*C*), the two PLVAP CC1 dimers from crystal form I are shown in side and top views. The small vertical green line denotes the position of a noncanonical heptad repeat (*defg*). (*D*) The PLVAP CC1 dimer from crystal form II is shown in side and top views.

**Fig. 7.**
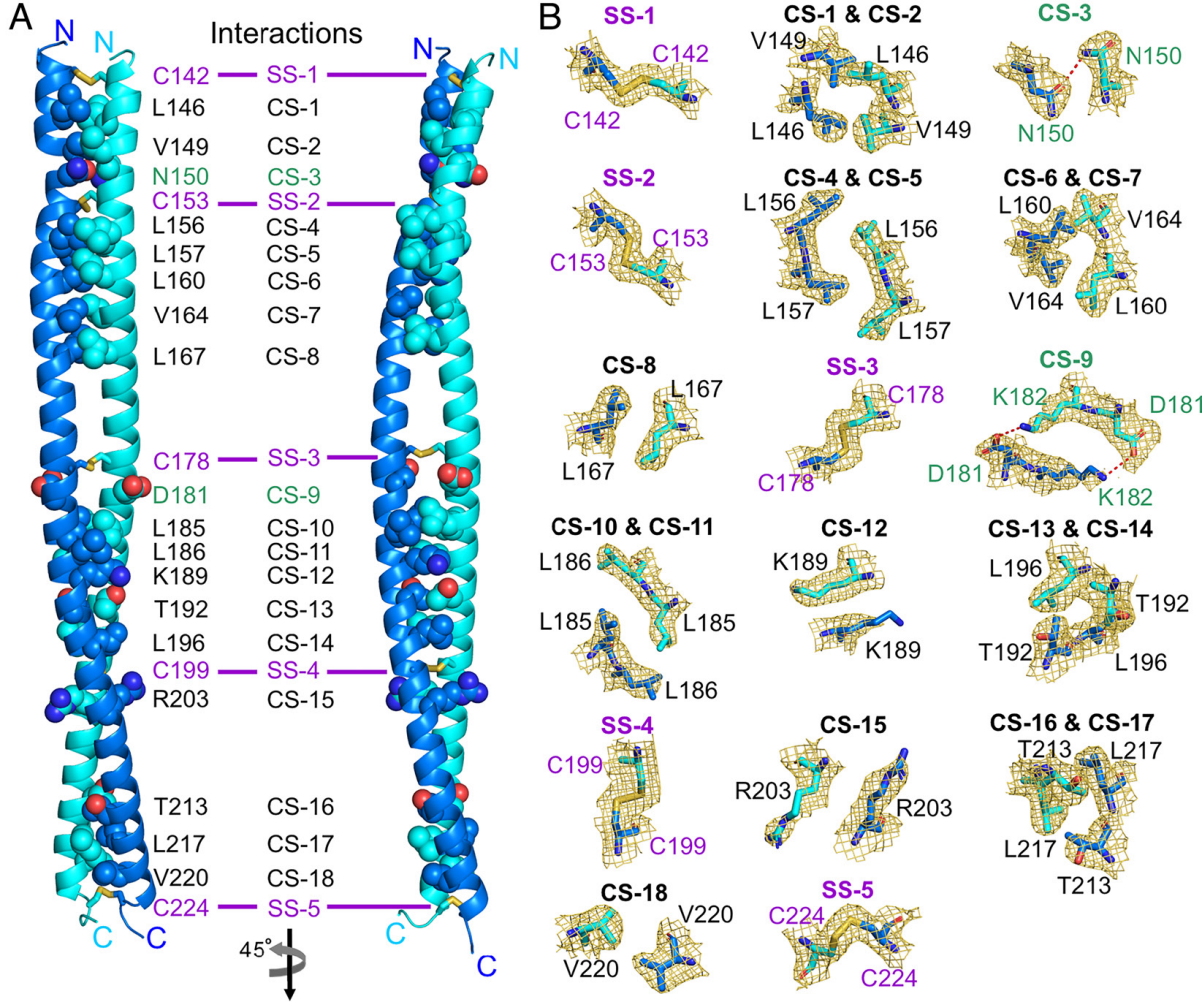
Interactions mediating PLVAP CC1 dimer formation. (*A*) Interactions for PLVAP CC1 dimer formation include (i) five disulfide bridges (SS-1 to SS-5) shown in stick representation, (ii) residues for hydrophilic interactions [cross-section (CS)-3 and CS-9], and (iii) hydrophobic interactions (multiple CSs) shown as sphere representations (atom coloring: carbon, marine, and cyan for each chain of the dimer, respectively; nitrogen, dark blue; oxygen, red). (*B*) Close-up views of the helix–helix interactions. The sigmaA-weighted 2|*F*_O_|-|*F*_C_| electron density map (golden meshes) is contoured at 1.0 σ, and residues for these interactions are shown in stick representation. Hydrophilic interactions are displayed as red dashed lines.

PLVAP CC1 is 89 amino acids in length and contains five cysteines (Fig. 6*A*). The structure of PLVAP CC1, determined from crystal form I by sulfur SAD, reveals a parallel dimer with five symmetric interchain disulfide bonds (SS-1 by Cys142, SS-2 by Cys153, SS-3 by Cys178, SS-4 by Cys199, and SS-5 by Cys224) (Fig. 6 *B* and *C*). The five disulfide bonds were confirmed in the anomalous difference map (Fig. 6 *B* and *C*). The PLVAP CC1 dimer model from crystal form I was used to determine the structure of PLVAP CC1 in crystal form II by MR (Fig. 6*D*). The electron density for residues 141 to 145 of crystal form II chain B was not visible (Fig. 6*D*). Structure-based analysis by the Socket2 algorithm (36) for both crystal forms shows that most of the PLVAP CC1 dimer exhibits a classical coiled-coil configuration characterized by heptad repeats (*abcdefg* in Fig. 6*A*). In addition to the five disulfide bonds, the PLVAP CC1 dimer interface features both hydrophobic and hydrophilic interactions (Fig. 7*A*). More specifically, there are sixteen hydrophobic interactions illustrated in cross-section (CS) in Fig. 7*B*: CS-1, Leu146; CS-2, Val149; CS-4, Leu156; CS-5, Leu157; CS-6, Leu160; CS-7, Val164; CS-8, Leu167; CS-10, Leu185; CS-11, Leu186; CS-12, Lys189 (using the proximal hydrophobic region of the side chain); CS-13, Thr192; CS-14, Leu196; CS-15, Arg203 (using the proximal hydrophobic region of the side chain); CS-16, Thr213; CS-17, Leu217; and CS-18, Val220. Two hydrophilic interactions are mediated by Asn150 (CS-3) and Asp181 and Lys182 (CS-9) (Fig. 7*B*).

The PLVAP CC1 heptad repeats present several exceptions to the classic coiled-coil pattern of hydrophobic amino acids at positions one (*a*) and four (*d*) that form the basis of knobs-in-holes packing (37, 38). In particular, Asn150, Asp181, Lys189, Thr192, Arg203, and Thr213 at positions *a* and *d* are hydrophilic (Fig. 6*A*). Asn150 contributes a hydrophilic interaction (CS-3), and the side chains of Lys189 (CS-12), Thr192 (CS-13), Arg203 (CS-15), and Thr213 (CS-16) form hydrophobic interactions that presumably stabilize the coiled-coil configuration (Fig. 7). Glu174 (position *d*), Gln206 (position *d*), and Gln210 (position *a*) do not appear to contribute to the coiled-coil configuration (Fig. 6*A*). The destabilizing effects of these noncanonical amino acids are presumably offset by the presence of disulfide bonds SS-3, SS-4, and SS-5 (Fig. 6 *C* and *D*). Last, a noncanonical heptad repeat (*defg*; resides 185 to 188; Fig. 6*A*) was identified by the Socket2 algorithm (36). Its destabilizing effect on the coiled-coil assembly is presumably offset by the presence of disulfide bonds SS-3 and SS-4 (Fig. 6*C*).

**Fig. 8.**
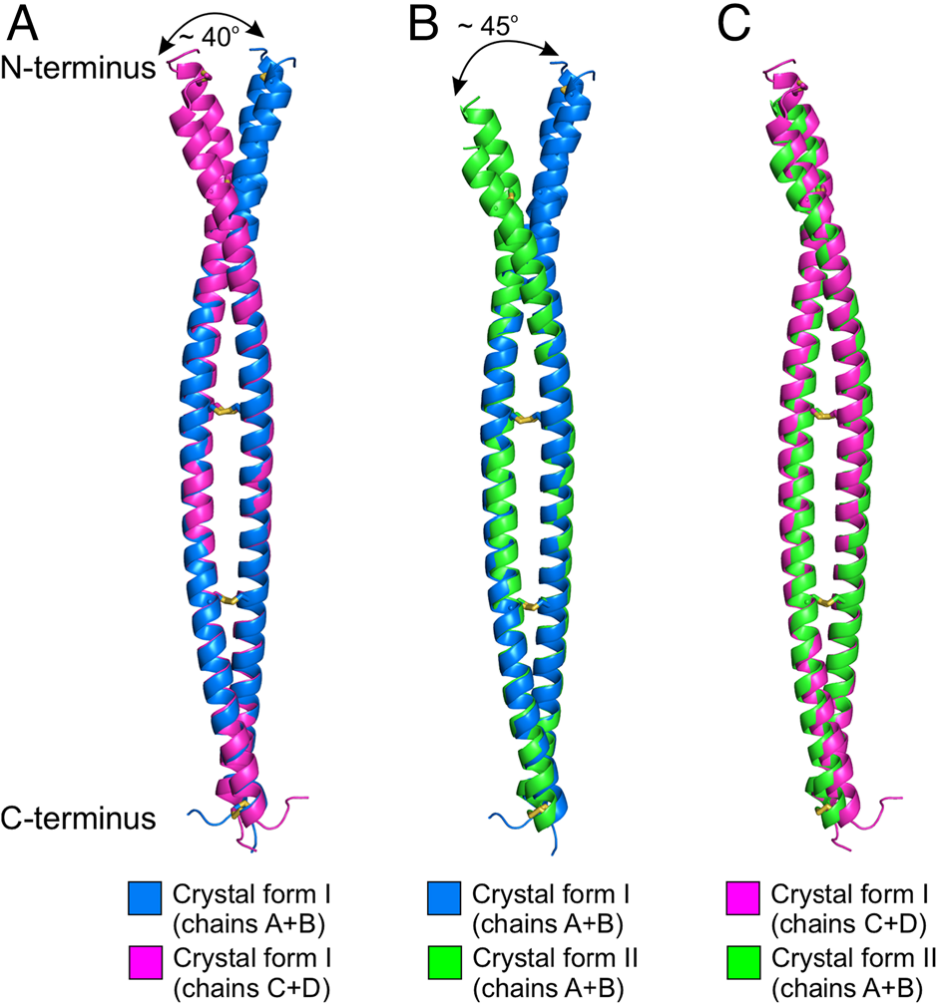
Structural comparisons between PLVAP CC1 dimers. (*A*) The superposition of chains A + B with chains C + D of PLVAP CC1 crystal form I has a rmsd of 1.15 Å over 139 Cα atoms. (*B*) The superposition of chains A + B of PLVAP CC1 crystal form I with chains A + B of crystal form II has a rmsd of 0.89 Å over 125 Cα atoms. (*C*) The superposition of chains C + D of PLVAP CC1 crystal form I and chains A + B of crystal form II has a rmsd of 0.84 Å over 150 Cα atoms.

Among PLVAP CC1 dimers, the average rmsds are 1.15 Å for crystal form I chain A + B vs. chain C + D, 0.89 Å for crystal form I chain A + B vs. crystal form II chain A + B, and 0.84 Å for crystal form I chain C + D vs. crystal form II chain A + B (Fig. 8). Conformational differences are greatest at the N terminus.

### Mapping the MECA-32 Epitope and Genetically Engineering MECA-32.

**Fig. 9.**
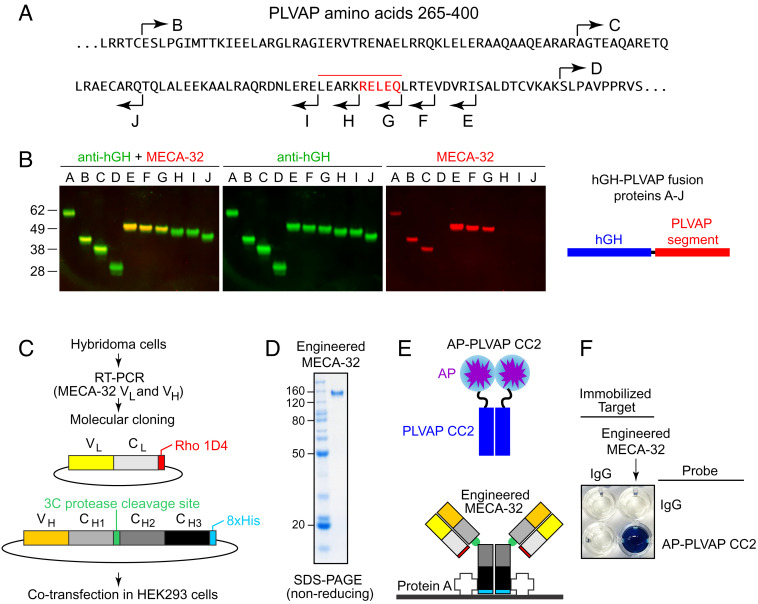
Mapping the epitope for MECA-32 on the PLVAP ECD. (*A*) The sequence of the PLVAP ECD between amino acids 265 and 400 showing the end points of deletions that remove progressively larger regions from the N terminus (rightward arrows *B*–*D*) and the end points of deletions that remove progressively larger regions from the C terminus (leftward arrows *E*–*J*). Amino acids essential for MECA-32 binding, as defined by PLVAP deletion analysis, are highlighted in red, with the overlying red bar indicating a larger region that likely encompasses the MECA-32 epitope. (*B*) SDS-PAGE and immunoblotting of hGH fusions with the PLVAP ECD deletions indicated in (*A*). Blots were probed with rabbit anti-hGH and mouse mAb MECA-32, and the primary antibodies were visualized with fluorescent secondary antibodies. Fusion proteins A-D extend to the C terminus of PLVAP. The N terminus of fusion protein A is at PLVAP amino acid 104 (TRREME...). Fusion proteins E-J have their N termini at PLVAP amino acid 143 (QEQLKE...). MECA-32 binds to fusion proteins A-C and E-G but not to fusion proteins D and H-J. (*C*) Workflow for MECA-32 Ab molecular cloning, construct design, and protein expression. (*D*) SDS-PAGE under nonreducing conditions of the engineered MECA-32 Ab (MECA-32/hIgG). (*E*) Diagram of the protein–protein interaction assay. MECA-32/hIgG was immobilized on protein A–coated wells. A fusion protein comprising alkaline phosphatase (AP) fused to PLVAP CC2 (AP–PLVAP CC2) was used as a probe. (*F*) A colorimetric AP reaction was used to detect the interaction between MECA-32/hIgG and AP–PLVAP CC2. Bovine IgG served as the negative control.

MECA-32, an anti-PLVAP mAb, is widely used as a marker of high-permeability vasculature (39, 40). Mapping the MECA-32 epitope and cloning and expressing recombinant MECA-32 could be of utility in the structural analysis of PLVAP and in the development of improved reagents for visualizing PLVAP in vivo. To map the MECA-32 epitope by immunoblotting, a series of deletion mutants of the PLVAP ECD were expressed as hGH fusion proteins (Fig. 9 *A* and *B*). Deletions from the PLVAP N terminus showed that the MECA-32 epitope is between amino acids 322 and 390, the interval defined by deletions C and D. A finer set of deletions from the PLVAP C terminus showed that the MECA-32 epitope is between amino acids 361 and 371, with an essential region between amino acids 367 and 371, the interval defined by deletions G and H.

To clone the variable domains of MECA-32, we reverse-transcribed and PCR amplified light-chain (V_L_) and heavy-chain (V_H_) segments from the MECA-32 hybridoma cell line (15) and inserted the PCR products into plasmids coding for a human light-chain constant region (C_L_ with a C-terminal rhodopsin 1D4 epitope tag) and a human heavy-chain IgG constant region (C_H1_, C_H2_, and C_H3_ with a human rhinovirus 3C protease cleavage site between C_H1_ and C_H2_ and a C-terminal 8xHis tag), respectively (Fig. 9*C*). The light and heavy chains encoded in the resulting constructs were coexpressed, assembled, and secreted from HEK/293T cells; we refer to this engineered protein as MECA-32/hIgG (Fig. 9*D*). The binding properties of MECA-32/hIgG were tested by immobilizing it on protein A–coated microwells, probing the wells with alkaline phosphatase (AP) fused to PLVAP CC2 (AP–PLVAP CC2), and detecting AP activity with a colorimetric enzyme assay (Fig. 9*E*). This assay shows strong and specific binding between MECA-32/hIgG and AP–PLVAP CC2 (Fig. 9*F*).

## Discussion

The three-dimensional structure of PLVAP CC1 presented here shows that it forms a parallel disulfide-bonded dimeric coiled coil. Additionally, our spectroscopic and biochemical studies of the PLVAP ECD and its subregions show that the entire PLVAP ECD adopts a predominantly alpha-helical conformation and that it assembles into a parallel disulfide-bonded, and presumably highly extended, dimer. These data lend strong support to the EC diaphragm model proposed by Tse and Stan (4) in which low–molecular weight blood and tissue substituents traverse the fenestral and vesicular diaphragms through wedge-shaped channels bounded on two sides by PLVAP dimers and on one side by the curved plasma membrane at the edge of the fenestra or vesicle (Fig. 1*C*). In this model, the lengths of the two longer sides of each opening are determined by the length of the PLVAP dimer, and the chemical properties of the channel walls are determined by the amino acid side chains and N-linked glycans (four conserved Asn-X-Ser/Thr are present; *SI Appendix*, Fig. S1*A*) on the solvent-accessible faces of PLVAP. The 64% estimate for the helical content of the ~390-amino acid PLVAP ECD—based on the CD spectrum in Fig. 2*E*—implies that ~248 amino acids are in a helical configuration. Assuming that the helices form a dimeric coiled coil with ~3.5 residues and ~5.1 Å per turn, then the helical part of the PLVAP ECD would extend ~361 Å (~36 nm), a close match to the ~40-nm radius of a diaphragm. It is interesting that, among mammals, the PLVAP ECD length is highly conserved (*SI Appendix*, Fig. S1*B*), consistent with the concept that coiled coils act as “molecular rulers” within macromolecular assemblies (37).

Fenestrated capillaries are present in a wide variety of locations, including endocrine organs, intestinal villi, renal peritubular capillaries, the choroid plexus, and circumventricular organs (1–3). In endocrine organs such as the pituitary, polypeptide hormones are secreted into the extracellular space and traverse the capillary wall, presumably via its fenestrations. The largest polypeptide hormones—thyroid-stimulating hormone, luteinizing hormone, and follicle-stimulating hormone—are heterodimeric glycoproteins of 28 to 35 kDa (41). Horse radish peroxidase (HRP), a 44-kDa protein, can also traverse EC diaphragms (42). In contrast, serum albumin, a nonglycosylated protein of 66 kDa, is unable to pass through EC diaphragms, as required for its role in maintaining serum oncotic pressure (11). It seems likely that, during evolution, these sieving properties of the EC diaphragm imposed a selective pressure to i) cap the molecular weights of polypeptide hormones at ~40 kDa and ii) increase the molecular weights of nonhormone serum proteins to greater than ~60 kDa. The selective pressure exerted on hormone and serum protein molecular weights was presumably mirrored by selective pressure on the sequence and structure of PLVAP so that it would produce EC diaphragms with an ~50-kDa cutoff.

Phase information is required for X-ray crystal structure determination, but it is lost during the collection of diffraction data (43–45). This “phase problem” is especially challenging for proteins that lack structurally defined homologs or accurate computational models because their structures cannot be solved by MR. In such cases, phase information is generally obtained by multiple isomorphous replacement (MIR) after soaking crystals with heavy atoms (e.g., gold, platinum, and mercury derivatives) or by MAD or SAD if an appropriate anomalous scatterer is present or can be introduced (43, 44, 46, 47). However, these approaches can be problematic (43, 48). For example, heavy atoms can degrade crystal quality or alter crystal packing so that the resulting crystal is no longer isomorphous (49), and SeMet incorporation often results in lower protein yield from eukaryotic cells, which can be problematic for proteins that are difficult to produce (50).

Sulfur SAD phasing is an attractive alternative for de novo phasing from native crystals, but this approach faces several technical challenges (23–26, 45, 51–55). First, the anomalous scattering signal from sulfur [atomic number (Z) = 16] is weak relative to scattering from selenium (Z = 34) and mercury (Z = 80). Second, the maximum anomalous signal is near the sulfur absorption edge at a wavelength of 5 Å, but such long-wavelength X-rays are associated with increased radiation damage, background absorption, and diffuse scattering and are generally unavailable at conventional synchrotron sources. Therefore, sulfur SAD data are typically collected at a wavelength of 1.5 to 2.5 Å with a synchrotron source (53) or at a wavelength of 2.29 Å with a chromium source (52). Third, the requirement for highly accurate sulfur SAD data requires the collection and subsequent merging of multiple datasets with minimal radiation damage during data collection (24, 25, 51, 56). To usefully merge sulfur SAD data from different crystals, the crystals must be isomorphous, and their point group symmetry must permit unambiguous indexing (57). Finally, the quality of sulfur SAD phasing is also dependent on the resolution of the diffraction data, with a resolution of 2.3 to 2.8 Å being generally desirable (24). Thus, collecting sulfur SAD data from poorly diffracting crystals has the added challenge that the higher X-ray dose required for useful diffraction leads to increased radiation damage.

In response to these challenges, several strategies have been developed. These include i) reducing air absorption with a vacuum or a helium-filled cone between the crystal and the detector (24, 55), ii) collecting diffraction data with an inverse-beam algorithm to minimize any differential effects of radiation damage on the signals from Friedel mates (25, 45), iii) using a semicylindrical detector to collect high-angle diffraction (55), and iv) using a high-precision multiaxis goniometer to collect multiple orientations from each crystal (26, 54, 55). At present, the instrumentation for implementing these strategies is available at only a few synchrotron facilities. A protein engineering strategy to enhance the utility of sulfur SAD involves adding rationally designed disulfide bonds (58).

In the present study, we combined a microfocused beam (~5 × 10^11^ photons s^−1^ with a beam cross-section of 5 μm × 7 μm) at a wavelength of 1.77 Å, a vector/helical data collection strategy, and an ultrafast shutter and high-sensitivity detector to collect 1,800° of sulfur SAD data from a single crystal in ~2.25 min. For comparison, Weinert et al. reported using a 90 μm × 45 μm beam cross-section with ~10^10^ photons s^−1^ at a wavelength of 2.07 Å to collect 2,880° (8 × 360°) of sulfur SAD data from a single crystal (26). We note that the Weinert et al.’s data collection required a multiaxis goniometer, an instrument that is not available in most synchrotron facilities (26).

In X-ray crystallography, structure determination of coiled-coil proteins has posed a long-standing challenge to the use of MR (22, 43, 59). MR is based on comparisons between the Patterson synthesis calculated from the model and the analogous synthesis generated from the diffraction data (43). Since coiled coils tend to pack in a parallel arrangement in the crystal, they generate many similar self- and cross-Patterson vectors that are difficult to disambiguate (22). Although recent advances in computational algorithms, including AMPLE (60), ARCIMBOLDO (61, 62), CCFold (33), and CCsolve (63), have made significant progress in structure determination of coiled-coil proteins by integrating computational modeling with MR, this strategy alone failed to solve the phase problem for PLVAP CC1.

As demonstrated here, the combination of protein modeling and MR with sulfur SAD is a powerful approach for structure determination (29–32). With MR–SAD, an initial phase calculation from either a full or partial MR difference Fourier analysis can be used to search for anomalous scattering peaks indicative of sulfur atoms. Importantly, the independence of MR and sulfur SAD phases minimizes the model bias that is inherent to the MR method. The MR–SAD strategy, together with technical advances in X-ray data collection, represents an important advance for structure determination of challenging targets.

## Materials and Methods

### Production and Purification of PLVAP ECD Fragments in *E coli*.

For expression in *E. coli*, a DNA segment coding for each PLVAP ECD fragment was cloned into a modified pET-11d vector (*SI Appendix*, Figs S2 *B* and S3 *B*). Starting from the N terminus, this vector codes for *E. coli* thioredoxin (*TrxA*), an 8xHis tag, and a 3C protease cleavage site (*SI Appendix*, Figs S2 *B* and S3 *B*). All constructs were confirmed by sequencing. The plasmid was transformed into *E. coli* SHuffle T7 cells (New England Biolabs) and induced with 0.2 mM isopropyl β-thiogalactopyranoside (IPTG) in terrific broth containing 100 μg/mL ampicillin (MilliporeSigma) at room temperature (~25^o^ C) overnight. For cell disruption, the cells were harvested by centrifugation and resuspended in B-PER bacterial protein extract reagent (ThermoFisher Scientific) supplemented with 50 mM HEPES, pH 7.5, 0.3 M NaCl, 30 mM imidazole, 1 mM MgCl_2_, 500 U benzonase (MilliporeSigma), 0.2 mg/mL lysozyme, and “cOmplete” Protease Inhibitor Cocktail (MilliporeSigma). The cell lysate was clarified by centrifugation, and the supernatant was filtered using a 0.45 μm Steritop filter (MilliporeSigma). Proteins were purified by immobilized metal affinity chromatography (IMAC) using Ni Sepharose 6 Fast Flow resin (Cytiva). The resin was washed with 20 mM HEPES, pH 7.5, 0.5 M NaCl, 30 mM imidazole, and10% glycerol and eluted in 20 mM HEPES, pH 7.5, 0.15 M NaCl, and 0.5 M imidazole. The eluted protein was dialyzed against 20 mM Tris, pH 7.5, 0.5 M NaCl, and 5% glycerol and treated with His-tagged 3C protease prepared as described previously (64). The 3C protease cleaved sample was further purified by IMAC and subjected to size-exclusion chromatography (SEC) using HiLoad Superdex 200 (Cytiva) in either 10 mM HEPES, pH 7.5, and 0.15 M NaCl or 10 mM Tris, pH 8.0, and 0.3 M NaCl.

### CD Spectra.

The four PLVAP ECD fragments (pXG52, pXG53, pXG61, and pPMS1170; Fig. 2*A*) were produced in *E. coli* SHuffle T7 cells as soluble thioredoxin fusions, released by 3C protease, and purified to apparent homogeneity as described above. Protein concentrations were determined by the Bradford assay and confirmed by Coomassie blue staining following SDS-PAGE, with a BSA dilution series as an internal standard. Stock solutions of highly concentrated protein (5 to 10 mg/mL) in 10 mM Tris, pH 8.0, 0.3 M NaCl (pXG52, pXG53, and pPMS1170) or 10 mM Tris, pH 7.0, and 0.4 M NaCl (pXG61) were dialyzed for 2.5 h against 10 mM Tris, pH 7.4, and 0.15 M NaCl, degassed for 30 min, and then diluted with dialysis buffer to a final protein concentration of 100 μM (pXG52, pXG53, and pPMS1170) or 40 μM (pXG61) for CD measurements.

Far-UV CD spectra were collected on a Jasco J-810 spectrophotometer. Spectra of 60 ul samples were recorded at 20°C using a 0.2-cm path-length cuvette and a 0.2-nm step size at a rate of one second per step. Spectra from the buffer control were subtracted from each protein’s spectrum. The data were then converted to molar residue ellipticity (MRE).

### Production and Immunoblotting of PLVAP Fragments Secreted from HEK/293T Cells.

For production of hGH fusion proteins in HEK/293T cells (ATCC CRL-11268), DNA segments coding for the PLVAP ECD or its fragments were inserted into the pSGHP1 expression vector (a derivative of pSGHV0; (65) 3′ of the hGH coding region and 8xHis tag). Adherent HEK/293T cells were transfected with polyethyleneimine (PEI) in the wells of a six-well tray, and the serum containing medium was replaced 1 d later with serum-free medium. After an additional day, the serum-free conditioned medium was collected, centrifuged 15 min at 3,000 rpm, and the supernatant stored in aliquots at −80 °C. For SDS-PAGE, serum-free conditioned medium was mixed with an equal volume of 2xSDS sample buffer either with or without beta-mercaptoethanol (BME) and heated to 90 °C for 3 min before loading. Immunoblots were probed with rat mAb MECA-32 (553849; BD Biosciences), rabbit polyclonal anti-hGH (RDI-HGHabrx1; Fitzgerald Industries International, Concord, MA), mouse mAb anti-rim (66), or rabbit polyclonal anti-myc as indicated in the figures. Blots were then probed with fluorescent goat anti-rabbit, anti-rat, or anti-mouse secondary antibodies (LI-COR Biosciences; Lincoln, Nebraska) and imaged with a LI-COR Odyssey Fc Imaging System.

### Crystallization and Data Collection.

PLVAP fragments CC1 and CC2 were concentrated to 10 mg/mL in 10 mM HEPES, pH 7.5, and 0.15 M NaCl and 12 mg/mL in 3 mM Bis-Tris, pH 6.5, and 90 mM NaCl for crystallization trials, respectively. Native crystals of PLVAP CC1 (space group *I*2_1_2_1_2_1_) were grown in 0.1 M sodium acetate, pH 5.0, and 15% polyethylene glycol (PEG) 4000 by the hanging drop vapor diffusion method at 21 °C. For sulfur SAD experiments, PLVAP CC1 crystals (space group *P*2_1_2_1_2_1_) were grown in 0.2 M sodium malonate, pH 5.0, and 20% PEG3350 by the sitting drop vapor diffusion method at 21 °C. PLVAP CC2 crystallized in 0.1 M MgCl_2_, 0.1 M MES, pH 6.0, and 8% PEG 6K at 21 °C. Crystals were transferred into a reservoir solution supplemented with 5 to 10% propane-1,2-diol for PLVAP CC1 and 25 to 30% glycerol for PLVAP CC2 and then cryocooled in liquid nitrogen.

Native diffraction data were collected at −173 °C on the 12-2 beamline with a PILATUS 6M detector (DECTRIS) at the Stanford Synchrotron Radiation Light Source at the National Accelerator Laboratory and were processed with the XIA2 system (67) with Diffraction Integration for Advanced Light Sources (DIALS) (68, 69). Two datasets were scaled and merged using AIMLESS (70). Because of severe anisotropic diffraction along the *h* axis of the reciprocal lattice, ellipsoidal and anisotropic scaling of diffraction data was performed using the STARANISO web server (71).

Sulfur SAD datasets were collected at −173 °C on the highly automated macromolecular crystallography (AMX) beamline in the National Synchrotron Light Source II (NSLS II) at the Brookhaven National Laboratory at a wavelength of 1.77 Å using an EIGER X 9M detector (DECTRIS) with the crystal-to-detector distance set to record reflections to 2.4 Å resolution at the corners of the square detector and to 2.6 Å resolution at the center of the detector’s sides. The vector module was used to collect continuous helical data over a 1,800° rotation with an exposure time of 0.015 s per 0.2° (with beam transmission attenuated to 10% from ~5 × 10^12^ photons s^−1^ to ~5 × 10^11^ photons s^−1^, with a beam cross-section of 5 μm × 7 μm). Images were indexed, integrated, and scaled using the XIA2 system (67) coupled with DIALS, POINTLESS, and AIMLESS (68–70, 72). A randomly selected subset of 5% of the diffraction data was used as a cross-validation dataset to calculate *R*_free_.

### Structure Determination and Refinement.

The structure of PLVAP CC1 was solved using sulfur SAD data from a 1,800° rotation of a single crystal (see preceding paragraph). Sulfur identification (substructure site) and initial anomalous signal analysis were conducted in the HKL2MAP interface (73) using SHELX C/D/E (74). Specifically, we tested various resolution cutoffs from 3.0 Å to 4.5 Å in steps of 0.5 Å with 1,000 trials per attempt using SHELX D searches. We used a resolution cutoff at 3.5 Å with 1,000 trials, Patterson search, and an *E*_min_ value of 1.5 to search for five disulfide bridges, resulting in 17 substructure sites. The substructures found in SHELX D were refined and completed for the final 24 substructure sites using PHASER (75) at PHENIX AutoSol (34, 35) with a resolution cutoff at 2.4 Å, and 4 noncrystallographic symmetry copies using THOROUGH for further substructure searches. The phases were calculated using PHASER (75) and then subjected to density modification using PHENIX RESOLVE (76) to generate an interpretable electron density map. Next, an initial model generated by PHENIX AutoBuild (35, 77) was manually rebuilt by COOT (78) and then fed into CCP4 BUCCANEER (79) and PHENIX Rosetta (35, 80) for further model building and geometry optimization. An anomalous difference Fourier map was calculated to assign the sulfur positions in the map for model building and structure validation. The native PLVAP CC1 structure with the space group *I*2_1_2_1_2_1_ was determined by MR using PHASER (75), with the structure of the PLVAP CC1 dimer obtained from the sulfur SAD data used as the template for MR.

For MR–SAD, the CCFold protein modeling algorithm (33) was used to generate a dimer model of PLVAP CC1, which was then used as a template for MR using PHASER (75). The resulting model and electron density map from PHASER (75) were entered into the MR–SAD pipeline in PHENIX AutoSol (34, 35) for anomalous difference Fourier analyses. An initial model generated by PHENIX AutoBuild (35, 77) was refined in PHENIX Refine (35). Manual model building in COOT and structure refinement in CCP4 REFMAC5 (81), PHENIX Refine (35), and autoBUSTER (82) were carried out iteratively for all structures. Translation–libration–screw (TLS) rotation was applied with noncrystallographic symmetry (NCS) restraints in all refinements. All models were validated with MOLPROBITY (83). The crystallographic statistics are listed in *SI Appendix*, Table S1.

### Bioinformatic Analysis and Graphic Presentation.

Structure-based multiple sequence alignment was performed using Clustal Omega (84) and ESPript (85). The secondary structure predictions for PLVAP were calculated using the Quick2D algorithm (17). Structure superposition was performed using SUPERPOSE (86) in the CCP4 suite (87). Structure-based coiled-coil analysis was performed using the Socket2 algorithm (36). High-quality images of the molecular structures were generated with the PyMOL Molecular Graphic System (version 2.5, Schrödinger, LLC). Schematic figures and other illustrations were prepared using CorelDRAW (Corel Corporation) and Illustrator (Adobe). Structural biology applications used in this work were compiled and configured by SBGrid (88).

### Sequencing MECA-32 mRNA and Generating Recombinant MECA-32/hIgG.

Total RNA from approximately 2 × 10^5^ MECA-32 hybridoma cells (15) was isolated using the RNeasy Plus Micro Kit (QIAGEN) and reverse-transcribed with random primers using SuperScript III Reverse Transcriptase (ThermoFisher Scientific). The variable domains of the heavy and light chains (V_H_ and V_L_) were PCR amplified from the resulting complementary DNA using the PCR primers described in ref. 89. Sequences of the PCR products showed the following protein sequences. Mature V_H_ (i.e., without the signal peptide): E​VQL​VES​GGG​LVQ​PGR​SMK​LSC​AAS​GFT​FSD​YYM​AWV​RQA​PMK​GLK​W​VAS​ISY​EGN​KTY​YGD​SVK​GRF​TIS​RDN​AKS​ILY​LQM​NSL​KSE​DTA​TYY​CAR​QSY​SSYIFDYWGQGVMVTVSS. Mature V_L_: D​IQM​TQT​PSS​MSA​SLG​ERV​TIS​CGT​SQG​VNI​FLN​WYQ​QKP​DGT​IKP​LIF​FTS​HLQ​SGV​PSR​FSG​SGS​GTA​YSL​TIS​SLE​PEDFAVYYCQQYDSSPPTFGGGTKLYLK. The PCR-amplified V_H_ and V_L_ segments were inserted into expression vectors containing a human heavy-chain IgG constant region (C_H1_, C_H2_, and C_H3_ with an HRV 3C protease cleavage site between C_H1_ and C_H2_ and a C-terminal 8xHis tag) and a human light-chain constant region [C_L_ with a C-terminal rhodopsin 1D4 epitope tag (90)], respectively (Fig. 9*C*). The heavy- and light-chain plasmids were cotransfected into HEK/293T cells, and the secreted MECA-32/hIgG was purified from conditioned media collected 2 d post-transfection by the IMAC method. For the human placental alkaline phosphatase (AP) fusion protein, DNA coding for PLVAP CC2 was cloned into the plasmid pHL-N-AP-Myc-8H which codes for AP followed by a Myc tag and an 8xHis tag (91). AP–PLVAP CC2 was expressed in HEK/293T cells. MECA-32/IgG was immobilized in the wells of protein A–coated 96-well plates (ThermoFisher Scientific) at 4 °C overnight. The wells were then washed three times with wash buffer (10 mM HEPES, pH 7.5, 0.15 M NaCl, and 0.05% Tween-20) and incubated with a 10-fold dilution of bovine serum albumin (BSA) blocking buffer (ThermoFisher Scientific) in wash buffer for 1 h at 25 °C. The wells were washed with wash buffer and incubated with conditioned media containing AP–PLVAP CC2 at 25 °C for 1 h. The wells were subsequently washed three times with wash buffer and incubated with BluePhos phosphatase substrate solution (Kirkegaard and Perry Laboratories) to visualize the bound AP probe.

## Supplementary Material

Appendix 01 (PDF)

## Data Availability

3D structure data have been deposited in [Protein Data Bank (PDB)] (8FBY and 8FCF) for PLVAP CC1 crystal forms I and II, respectively (92, 93).
