# Supplementary material for: Structural insights into plasmalemma vesicle-associated protein (PLVAP): Implications for vascular endothelial diaphragms and fenestrae
Source: Proc Natl Acad Sci U S A. 2023 Mar 30;120(14):e2221103120. doi: 10.1073/pnas.2221103120 (PMC10083539; doi:10.1073/pnas.2221103120)
Supplement: Supplementary file 1 — Appendix 01 (PDF) [file pnas.2221103120.sapp.pdf]

Supplementary information for

Biochemical and structural insights into Plasmalemma Vesicle-Associated Protein (PLVAP): implications for vascular endothelial diaphragms and fenestrae

Tao-Hsin Chang[1,7,*], Fu-Lien Hsieh[1,7], Xiaowu Gu[1,7,†], Philip M. Smallwood[1,7], Jennifer Kavran[2,3,8], Sandra B. Gabelli[2,3,4,‡], Jeremy Nathans[1,5,6,7,*]

[1]Department of Molecular Biology and Genetics
[2]Department of Biophysics and Biophysical Chemistry
[3]Department of Oncology
[4]Department of Medicine
[5]Department of Neuroscience
[6]Wilmer Eye Institute
[7]Howard Hughes Medical Institute
Johns Hopkins University School of Medicine, Baltimore, Maryland, United States.

[8]Department of Biochemistry and Molecular Biology,
Bloomberg School of Public Health, Johns Hopkins University, Baltimore, Maryland, United States.

*Corresponding authors

†Present address: Genentech, 1 DNA Way, South San Francisco, CA 94080

‡Present address: Merck & Co., Inc., 770 Sumneytown Pike, West Point, PA 19486

Running title: PLVAP structure

Keywords: vasculature, permeability, alpha helix, coiled-coil, sulfur SAD

*Addresses for editorial correspondence:
Dr. Jeremy Nathans; Dr. Tao-Hsin Chang
805 PCTB
725 North Wolfe Street
Johns Hopkins University School of Medicine
Baltimore, MD 21205
tel: 410 955 4679
fax: 410 614 0827
email: jnathans@jhmi.edu; taohsin.chang@gmail.com

References

1. Sievers F & Higgins DG (2014) Clustal omega. *Curr Protoc Bioinformatics* 48:3 13 11-16.
2. Robert X & Gouet P (2014) Deciphering key features in protein structures with the new ENDscript server. *Nucleic Acids Res* 42(Web Server issue):W320-324.
3. Pape T, Schneider, Thomas R., (2004) HKL2MAP: a graphical user interface for macromolecular phasing with SHELX programs. *Journal of Applied Crystallography* 37:843-844.
4. Sheldrick GM (2010) Experimental phasing with SHELXC/D/E: combining chain tracing with density modification. *Acta Crystallogr D Biol Crystallogr* 66(Pt 4):479-485.
5. Guzenko D & Strelkov SV (2018) CCFold: rapid and accurate prediction of coiled-coil structures and application to modelling intermediate filaments. *Bioinformatics* 34(2):215-222.
6. Gonzalez JE & Tsien RY (1997) Improved indicators of cell membrane potential that use fluorescence resonance energy transfer. *Chemistry & biology* 4(4):269-277.
7. Karplus PA & Diederichs K (2012) Linking crystallographic model and data quality. *Science* 336(6084):1030-1033.

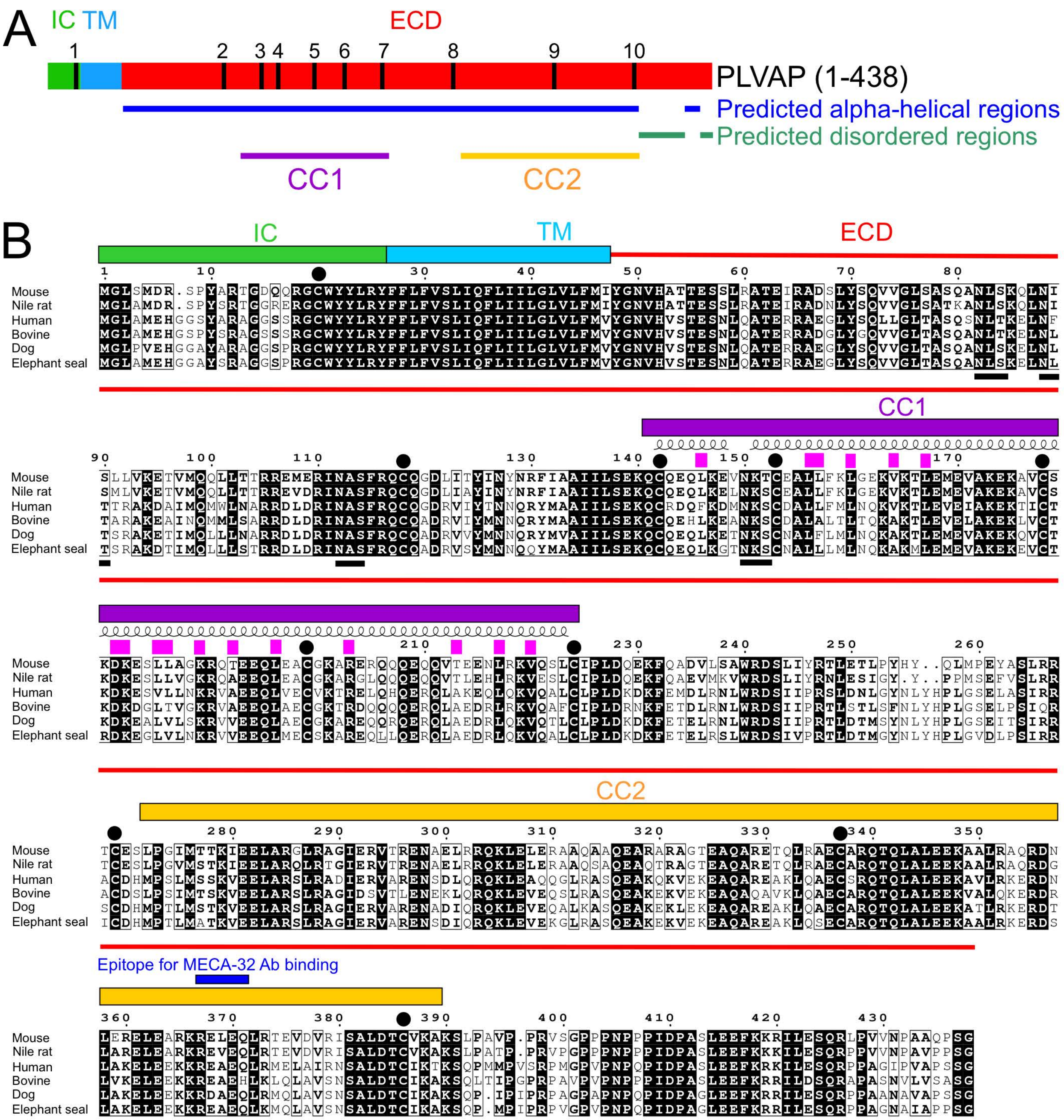

Fig. S1. PLVAP and its multiple sequence alignment. (A) Schematic diagram of mouse PLVAP containing residues 1 to 438 (IC, intracellular domain; TM, transmembrane domain; ECD, extracellular domain; CC1, coiled-coil domain 1; CC2, coiled-coil domain 2). Ten cystine residues are denoted as black lines and numbered 1 to 10. Predicted alpha-helical and disordered regions (amino acids 390-409 and 425-438) are indicated by blue and green lines, respectively. (B) Protein sequences of PLVAP from Mouse (NCBI accession: EDL28929), Nile rat (NCBI accession: XM_034519814.1); Human (NCBI accession: NP_112600.1), Bovine (NCBI accession: NP_001030430.1), Dog (NCBI accession: XP_038284168.1), and Elephant seal (NCBI accession: XM_034997037.1) are aligned using Clustal Omega (1) and formatted using ESPript (2). Secondary structure elements and numbering for mouse PLVAP CC1 are shown above the sequences. The positions of cysteines are indicated by black circles. Four putative N-linked glycosylation sites (Asn-X-Ser/Thr; X for any residue) are indicated by black

underlines. Residues contributing to the coiled-coil formation of CC1 except the five disulfide bonds are denoted by magenta rectangles (see also **Fig. 7**). The epitope on PLVAP CC2 for MECA-32 Ab binding is denoted by a blue rectangle.

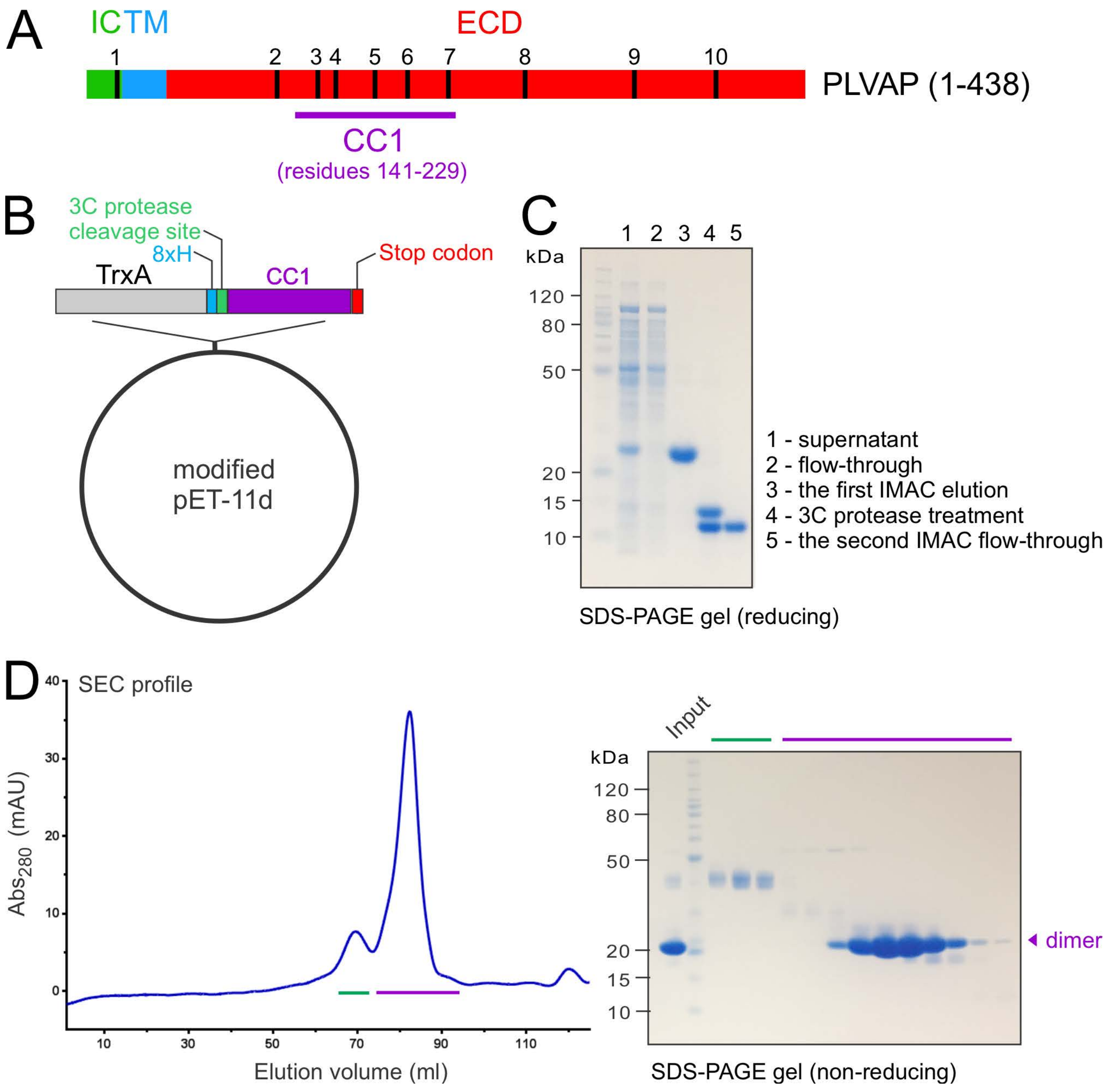

Fig. S2. Protein expression and purification of mouse PLVAP CC1 from *E. coli* Shuffle cells. (A) Schematic diagram of mouse PLVAP (see Fig. S1 for abbreviations). A DNA fragment coding PLVAP CC1 (residues 141 to 229) was inserted in a modified pET-11d vector. (B) The modified pET- 11d vector (pPMS-1170) has the coding region for *E. coli* thioredoxin (*TrxA*) followed by an 8xHis tag and a 3C protease cleavage site. (C) Coomassie-stained SDS-PAGE run under reducing conditions shows the results of IMAC purification and 3C protease cleavage of the TrxA-CC1 fusion protein. (D) SEC elution profile and SEC fractions were analyzed by Coomassie-stained SDS-PAGE under non-reducing conditions. The green line corresponds to contaminated fractions and the purple line corresponds to PLVAP CC1 dimer fractions.

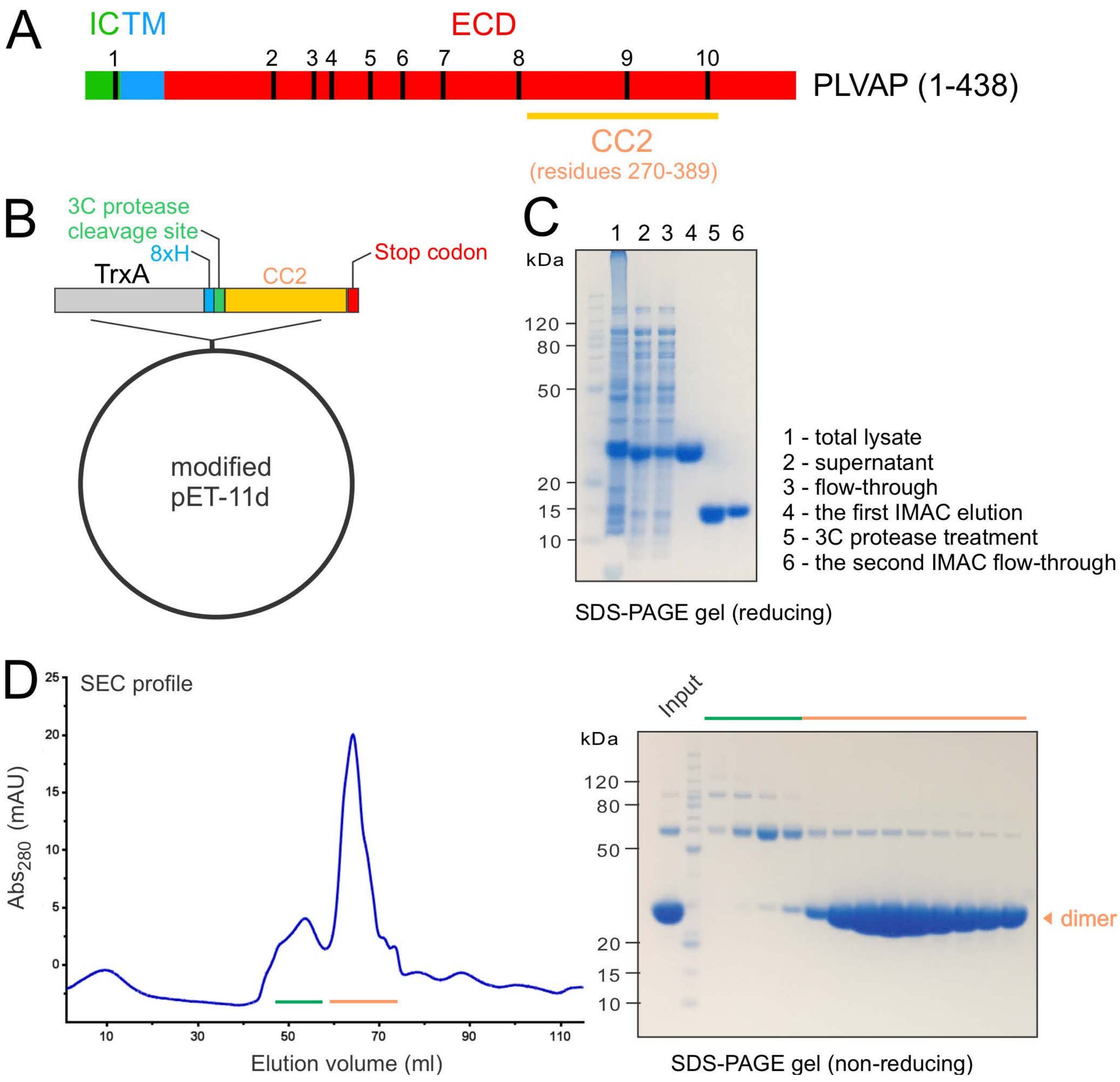

Fig. S3. Protein expression and purification of mouse PLVAP CC2 from *E. coli* Shuffle cells. (A) Schematic diagram of mouse PLVAP (see Fig. S1 for abbreviations). A DNA fragment coding PLVAP CC2 (residues 270 to 389) was inserted in a modified pET-11d vector. (B) The modified pET- 11d vector (pPMS-1573) has the coding region for *E. coli* thioredoxin (*TrxA*) followed by an 8xHis tag and a 3C protease cleavage site. (C) Coomassie-stained SDS-PAGE run under reducing conditions shows the results of IMAC purification and 3C protease cleavage of the TrxA-CC2 fusion protein. (D) SEC elution profile and SEC fractions were analyzed by Coomassie-stained SDS-PAGE under non-reducing conditions. The green line corresponds to contaminated fractions and the orange line corresponds to PLVAP CC2 dimer fractions.

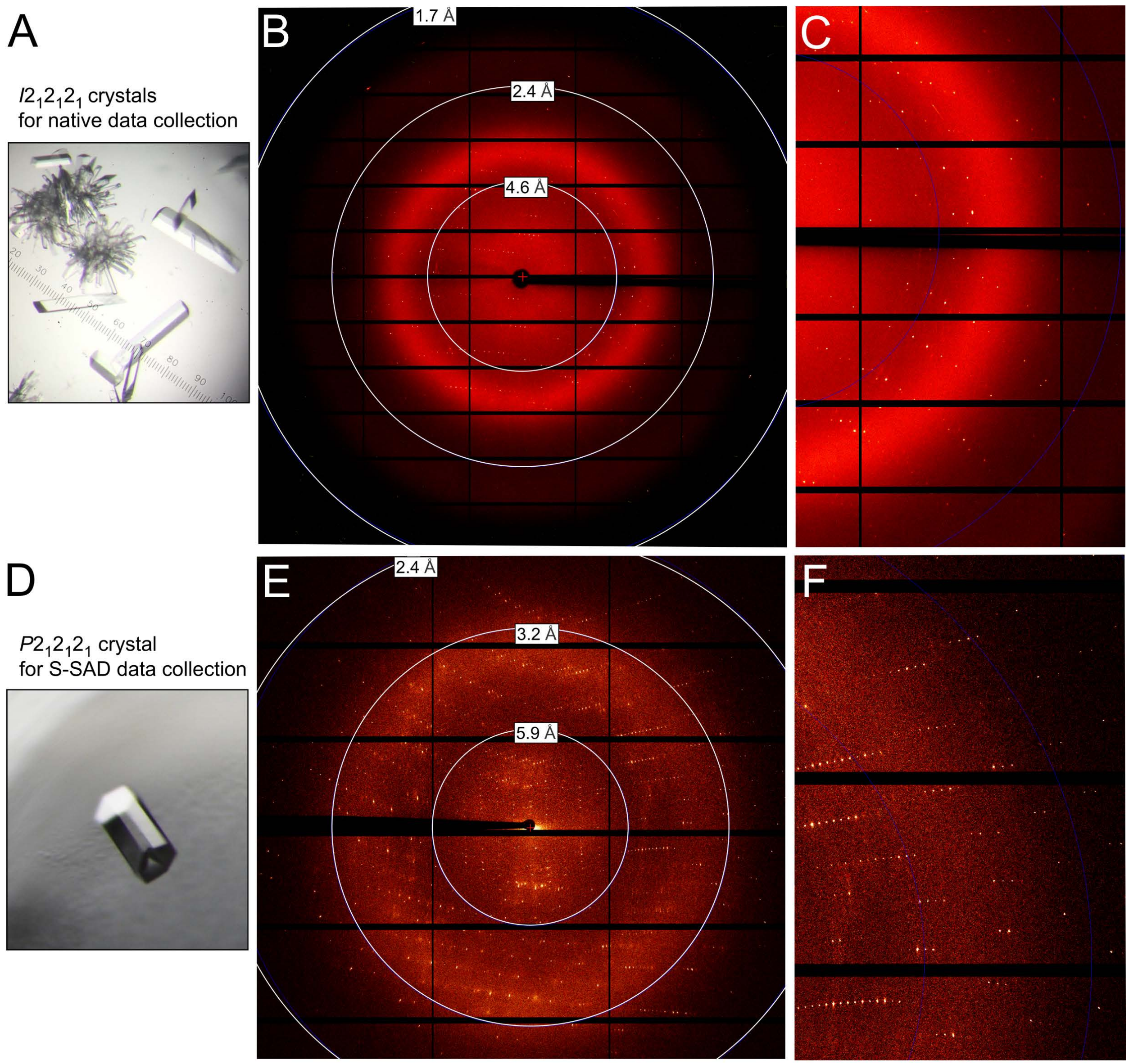

Fig. S4. Crystals and X-ray diffraction images of PLVAP CC1. (A) PLVAP CC1 crystals in the $I2_12_12_1$ space group (crystal form II) used for the native data collection on the 12-2 beamline (SSRL). (B) An X-ray diffraction image of PLVAP CC1 crystal form II collected using a PILATUS 6M detector. (C) A close-up view of (B) beyond the resolution shell of 4.6 Å. (D) A typical PLVAP CC1 crystal in the $P2_12_12_1$ space group (crystal form I) used for S-SAD data collection on the AMX beamline (NSLS II). (E) An X-ray diffraction image of PLVAP CC1 crystal form I collected using an EIGER X 9M detector. (F) A close-up view of (E) beyond the resolution shell of 3.2 Å.

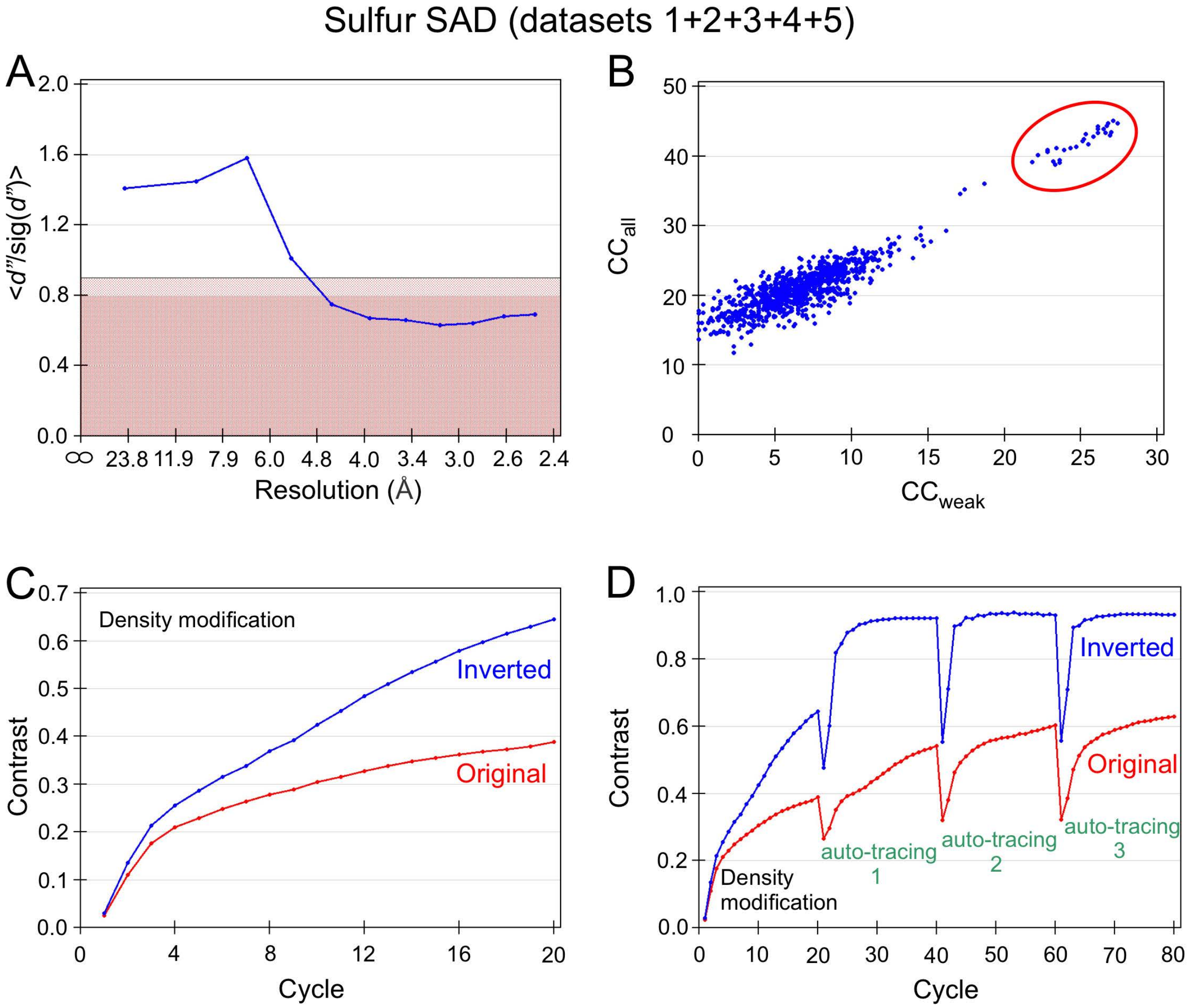

Fig. S5. Anomalous sulfur phasing using five datasets from a single crystal. (A) The *HKL2MAP* (3) profile of *d"*/sig(*d"*) versus X-ray diffraction resolution shells reveals the signal-to-noise ratio of the anomalous differences. The red part of the graph indicates where the anomalous signal is extremely weak or negligible. (B) The *HKL2MAP* profile of correlation coefficients between observed and calculated Bijvoet differences shown by CC_{all} versus CC_{weak}. Successful solutions are indicated by a red circle. (C) The *HKL2MAP* profile shows contrast versus cycle number for the density modification with phases calculated using the original hand (red) or inverted hand (blue) substructure. (D) The *HKL2MAP* profile of contrast versus cycle number for the density modification and chain tracing using *SHELXE* (4), based on the original hand (red) or inverted hand (blue) substructure.

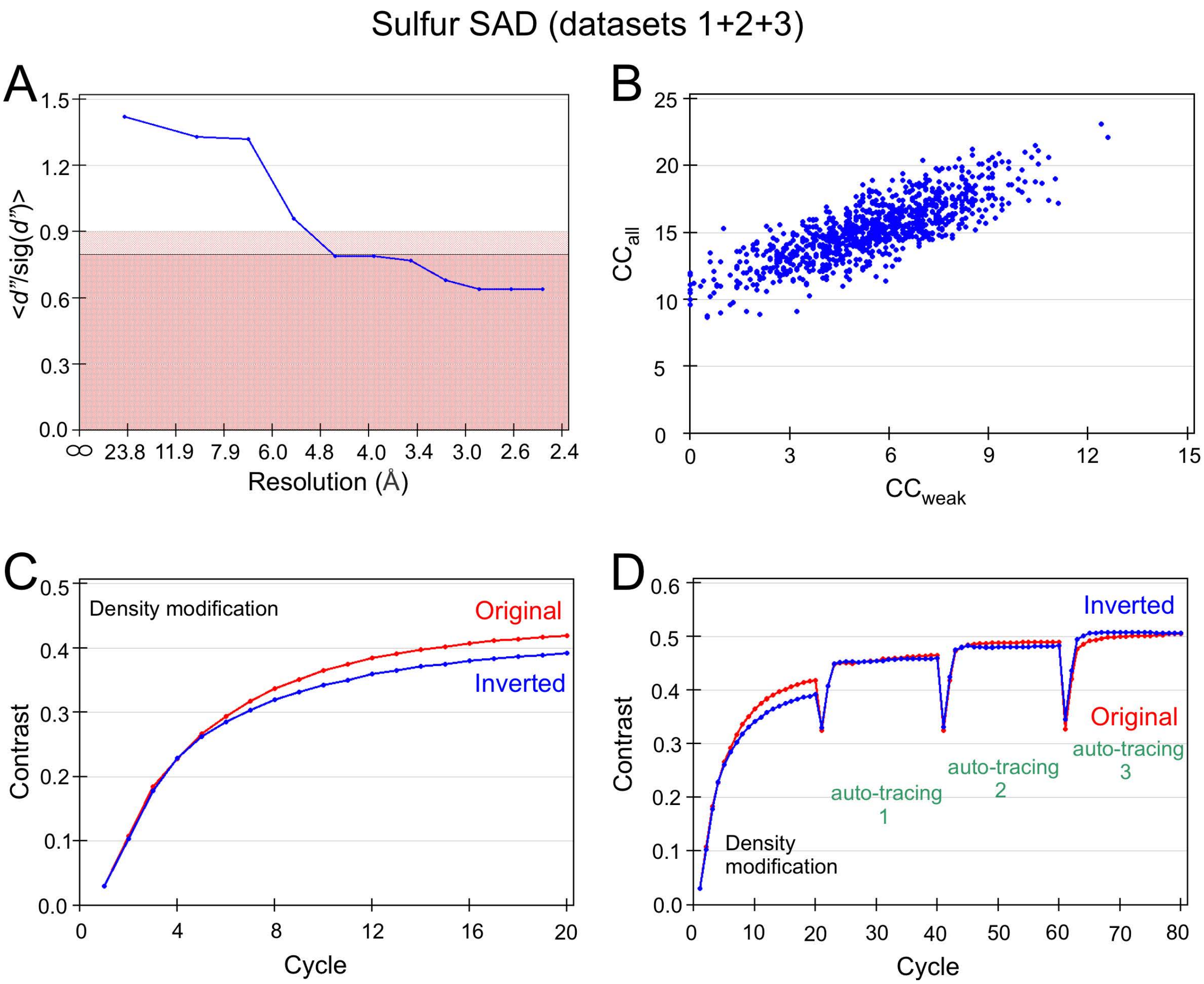

Fig. S6. Anomalous sulfur phasing using three datasets from a single crystal. (A) The *HKL2MAP* plot of *d''*/sig(*d''*) versus X-ray diffraction resolution shells. (B) The *HKL2MAP* plot reveals the distribution of CC_{all} and CC_{weak} values. (C) The *HKL2MAP* plot of contrast versus cycle number for the density modification using the original hand (red) or inverted hand (blue) substructure. (D) The *HKL2MAP* plot shows contrast versus cycle number for the density modification and chain tracing using *SHELXE* (4).

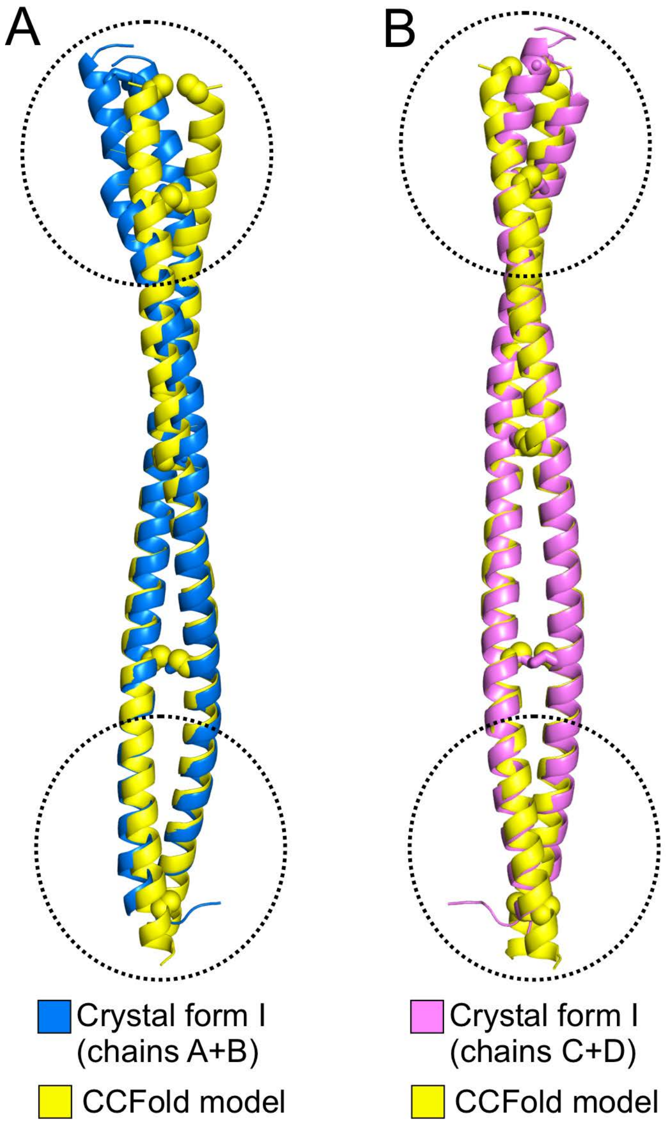

Fig. S7. Structural comparisons of PLVAP CC1 structure (crystal form I) and the computational model of PLVAP CC1 generated by the CCFold algorithm (5). (A) Superposition of chains A+B of PLVAP CC1 crystal form I and the CCFold model has a r.m.s. deviation of 1.58 Å over 146 Cα atoms. (B) Superposition of chains C+D of PLVAP CC1 crystal form I and the CCFold model has a r.m.s. deviation of 1.42 Å over 152 Cα atoms.

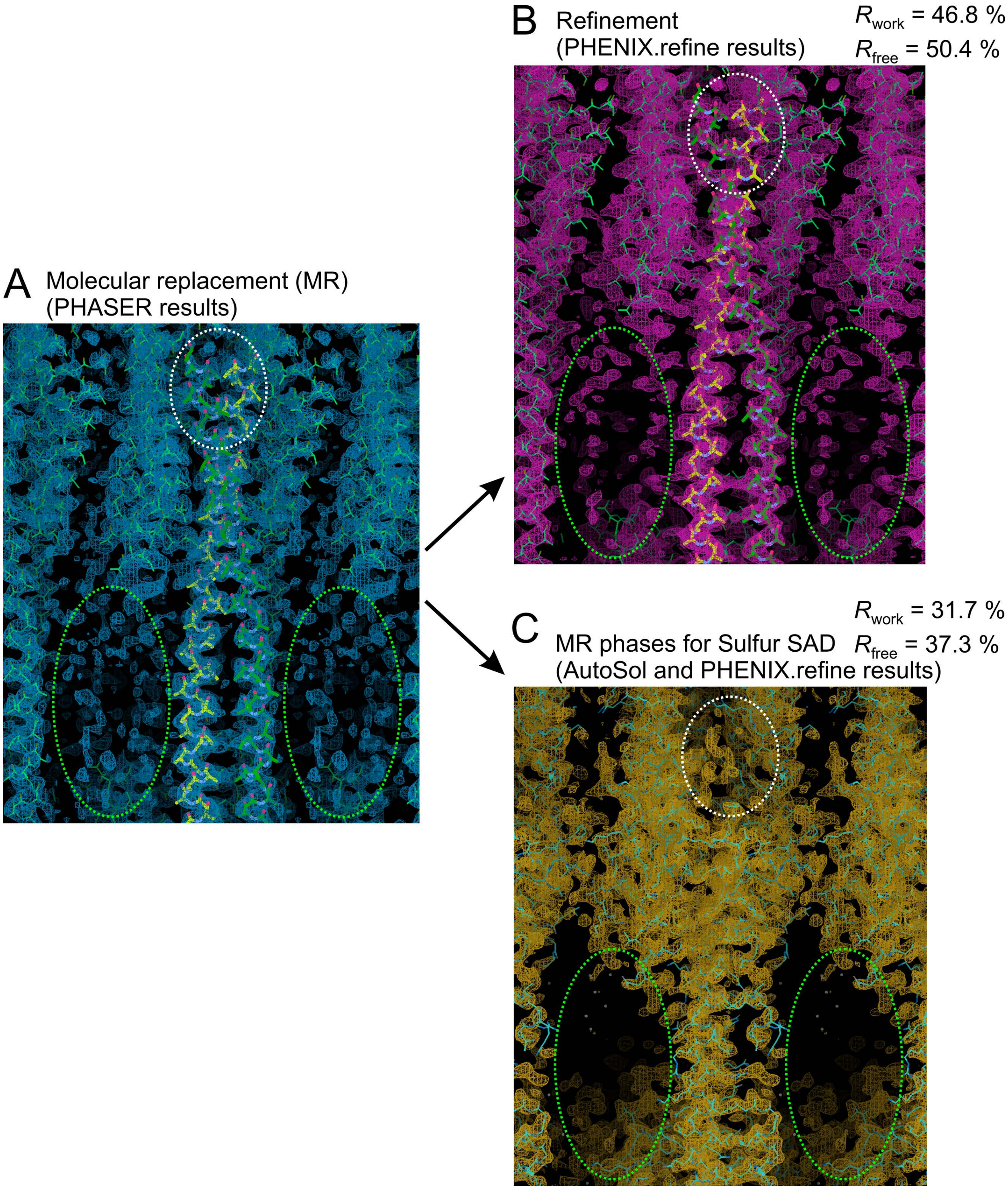

Fig. S8. MR combined with Sulfur SAD for structure determination of PLVAP CC1 using three datasets from a single crystal. (A) The initial electron density map (blue meshes) is contoured at 1.5 σ after MR using the computational model of the PLVAP CC1 dimer generated by CCFold (5) as the search model. The major solvent regions are denoted by green dashed circles. The white dashed circle indicates one of the regions where the model is placed incorrectly, as determined by comparing with (C). (B) The electron density map (purple meshes) contoured at 1.5 σ after refinement starting with MR solution shown in (A).

The high values for R_{factor} (46.8%) and R_{free} (50.4%) indicate that the MR solution shown in (A) has low quality phase information. (C) MR phases were modified with phase information calculated using three Sulfur SAD datasets. The electron density map (golden meshes) contoured at 1.5 σ shows that the combined phases calculated by MR and Sulfur SAD leads to a refined structure with R_{factor}=31.7% and R_{free}=37.3%. The three Sulfur SAD datasets alone do not lead to a correctly refined structure (**Fig. S6**).

Table Supplement 1. Data collection and refinement statistics.

	S-SAD	Native[a]
Crystal form	I	II
**Data collection**		
Space group	$P2_12_12_1$	$I2_12_12_1$
Unit-cell parameters		
a, *b*, *c* (Å)	35.6, 86.2, 181.6	33.8, 89.0, 180.9
α, β, γ (°)	90.0, 90.0, 90.0	90.0, 90.0, 90.0
No. of crystals	1	2
Wavelength	1.77122	0.97946
Resolution (Å)	90.82 - 2.40 (2.44 - 2.40)	39.93 - 1.95 (2.05 - 1.95)
No. of unique reflections	22760 (1031)	14322 (754)
Completeness (%)	99.9 (94.2)	93.0 (88.0)
Multiplicity	60.4 (36.4)	12.1 (10.8)
I / σ (*I*)	29.6 (10.2)	8.7 (2.8)
R_{pim} (%)[b]	1.6 (5.8)	4.1 (32.9)
$CC_{1/2}$ (%)[c]	99.8 (99.2)	99.5 (80.75)
**Refinement**		
Resolution (Å)	90.82 - 2.40	39.93 - 1.95
R_{work} / R_{free} (%)	22.3 / 26.1	24.5 / 27.8
No. atoms		
Protein	2826	1287
Water	181	93
B-factors		
Protein	58.7	44.0
Water	56.4	41.6
R.m.s deviations[d]		
Bond lengths (Å)	0.009	0.012
Bond angles (°)	1.17	1.48
Ramachandran plot		
Favored (%)	97.7	100.0
Allowed (%)	2.3	0.0
PDB code		

[a] The X-ray diffraction data were merged from two crystals.
[b] R_{pim}: Precision-indicating merging R-factor (6).
[c] $CC_{1/2}$: correlation coefficients between random half data sets (7).
[d] R.m.s deviations: root mean square deviation from ideal geometry.
Values in parentheses are for highest-resolution shell.
